# Supplementary material for: Modeling heterogeneous signaling dynamics of macrophages reveals principles of information transmission in stimulus responses
Source: Nat Commun. 2025 Jul 1;16:5986. doi: 10.1038/s41467-025-60901-3 (PMC12218168; doi:10.1038/s41467-025-60901-3)
Supplement: Supplementary file 1 — Supplementary Information [file 41467_2025_60901_MOESM1_ESM.pdf]

Part I: Supplementary Figures

Modeling single-cell heterogeneity in signaling dynamics of macrophages reveals principles of information transmission

Xiaolu Guo[1,2], Adewunmi Adelaja[1,2,3], Apeksha Singh[1,2], Roy Wollman[1,4], Alexander Hoffmann*[,1,2]

[1] Institute for Quantitative and Computational Biosciences, University of California Los Angeles, Los Angeles, USA
[2] Department of Microbiology, Immunology, and Molecular Genetics, University of California Los Angeles, Los Angeles, USA
[3] Current address: Harvard combined Dermatology Residency Training Program, Boston, MA, USA
[4] Department of Integrative Biology and Physiology, University of California Los Angeles, Los Angeles, USA

* Corresponding author: ahoffmann@ucla.edu

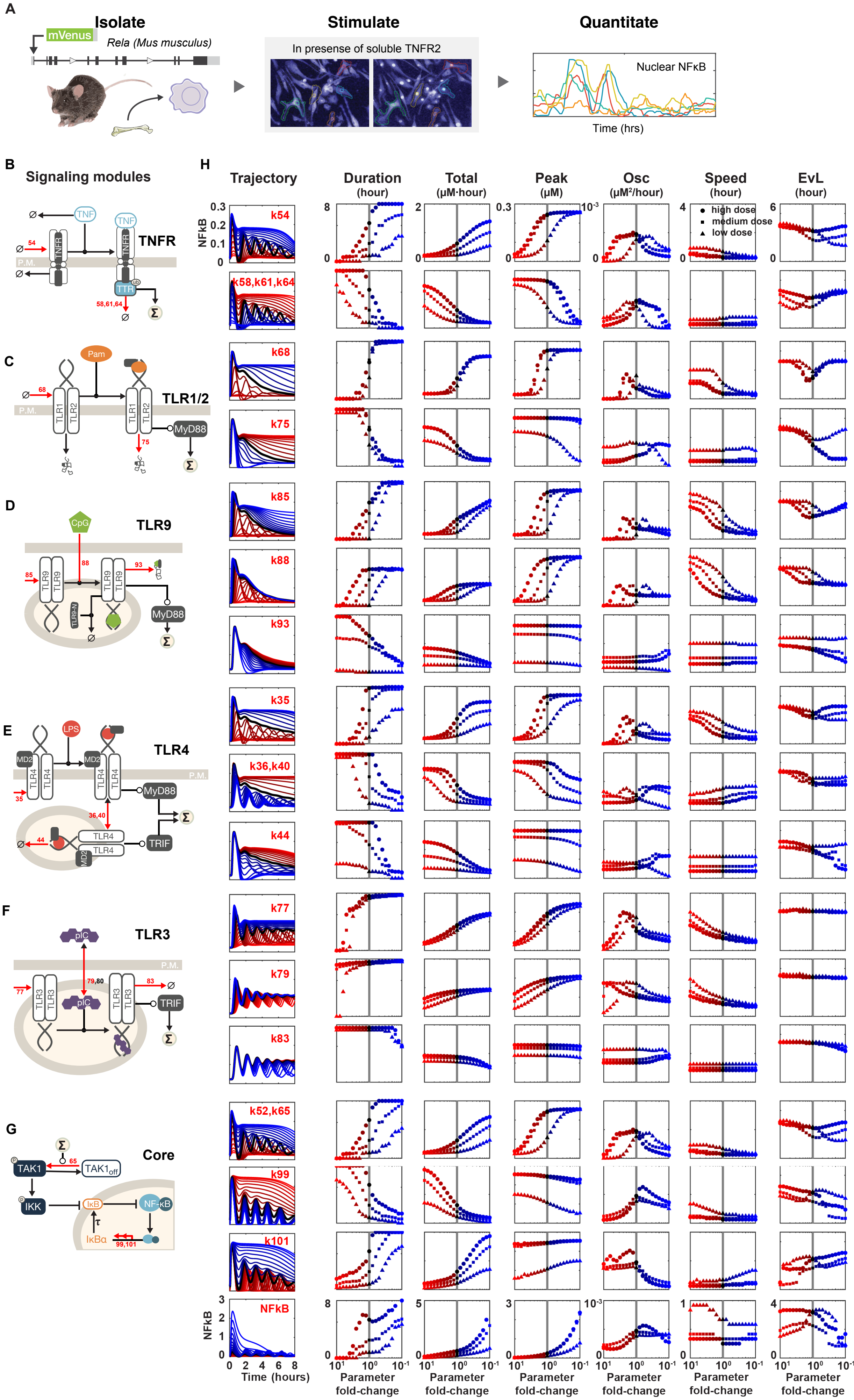
A
Isolate
mVenus
Rela (Mus musculus)
Stimulate
In presense of soluble TNFR2
Quantitate
Nuclear NFκB
Time (hrs)
B
Signaling modules
TNF
TNFR
TTR
P.M.
C
Pam
TLR1/2
TLR1
TLR2
MyD88
D
CpG
TLR9
MyD88
E
LPS
TLR4
MD2
MyD88
TRIF
F
pIC
TLR3
TRIF
G
Core
TAK1
TAK1off
IKK
IκB
NF-κB
IκBα
H
Trajectory
Duration (hour)
Total (μM·hour)
Peak (μM)
Osc (μM²/hour)
Speed (hour)
EvL (hour)
high dose
medium dose
low dose
NFkB
k54
k58,k61,k64
k68
k75
k85
k88
k93
k35
k36,k40
k44
k77
k79
k83
k52,k65
k99
k101
NFkB
Time (hours)
Parameter fold-change

Figure S1. Workflow of experiments and sensitivity analysis for selected parameters within NFκB signaling network.

A. Schematic of experimental workflow: Bone marrow-derived macrophages (BMDMs) from knockin mice expressing mVenus-RelA from the endogenous RelA locus were imaged under various stimulus conditions that block auto-or paracrine signaling by TNF. Nuclear NFκB levels in single cells over time were quantified by automatic image analysis and fluorescence quantification. Adapted from Adelaja et al. (2021) [1] (https://www.cell.com/immunity/fulltext/S1074-7613(21)00173-4): the stimulate step have been updated to include the presence of soluble TNFR2.

B-G. Schematics of indicated signaling modules. Selected parameters include receptor synthesis rates (k54 for TNFR, k68 for TLR1/2, k85 for TLR9, k35 for TLR4, k77 for TLR3), endosomal import rates (k88 for CpG module, k36 and k40 for LPS module, k79 for pIC module), and signaling complex degradation rates for receptor modules (k58, k61, and k64 for TNF module, k75 for Pam module, k44 for LPS module, k83 for pIC module), , and TAK1 activation rate (k65), time delay for the NFκB-regulated IκBα transcription (k99 and k101), and total NFκB concentration for core module. These parameters are marked in red font with the reaction number labeled. Adapted from Adelaja et al. (2021) [1] (https://www.cell.com/immunity/fulltext/S1074-7613(21)00173-4): the panels have been updated to include enough details of biochemical reactions, with the red arrows representing the parameters for sensitivity analysis.

H. Parameter sensitivity analysis for the selected parameters within each module, as specified in (A-F). Effect of variations of parameter(s) on the dynamic trajectory of NFκB activity (first column), or on NFκB dynamic trajectory features that were shown to be informative of the stimulus (Adelaja et al 2021) and specified in methods (second to seventh columns). The y-axis scales of all the plots in the same column are the same as the first row, except the last row as specified. The x-axis scales are the same and are specified in the last row.

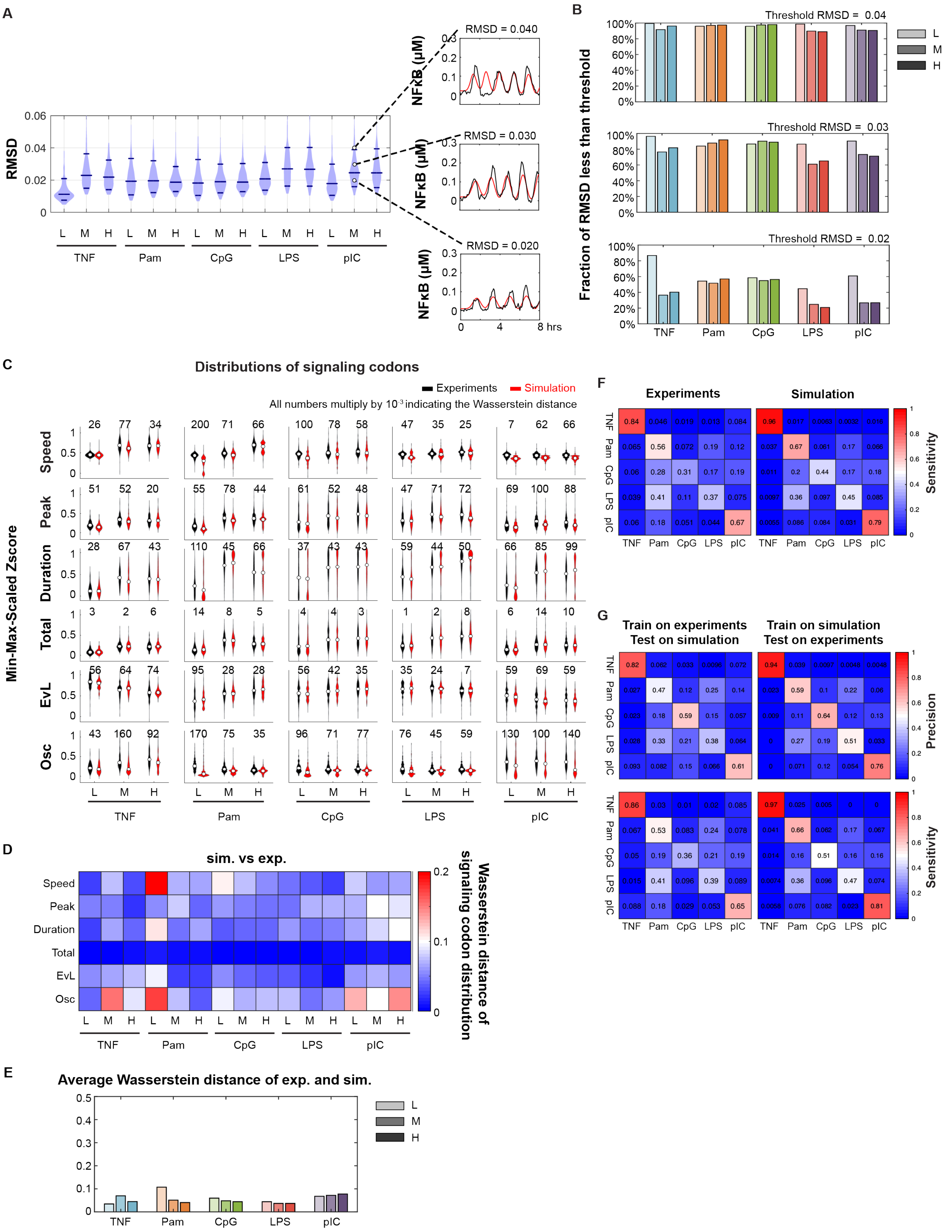
A
RMSD
TNF
Pam
CpG
LPS
pIC
L
M
H
RMSD = 0.040
RMSD = 0.030
RMSD = 0.020
NFκB (μM)
8 hrs
B
Fraction of RMSD less than threshold
Threshold RMSD = 0.04
Threshold RMSD = 0.03
Threshold RMSD = 0.02
C
Distributions of signaling codons
Experiments
Simulation
All numbers multiply by 10^-3 indicating the Wasserstein distance
Min-Max-Scaled Zscore
Speed
Peak
Duration
Total
EvL
Osc
D
sim. vs exp.
Wasserstein distance of signaling codon distribution
E
Average Wasserstein distance of exp. and sim.
F
Experiments
Simulation
Sensitivity
G
Train on experiments Test on simulation
Train on simulation Test on experiments
Precision
Sensitivity

H

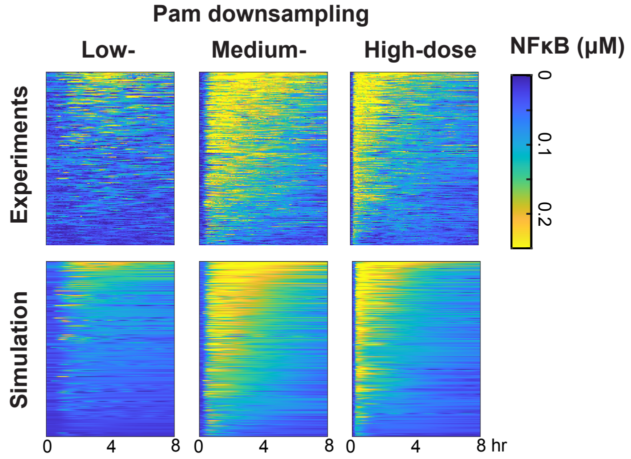

Pam downsampling
Low-
Medium-
High-dose
NFκB (μM)
Experiments
Simulation
0
0.1
0.2
0 4 8 0 4 8 0 4 8 hr

I

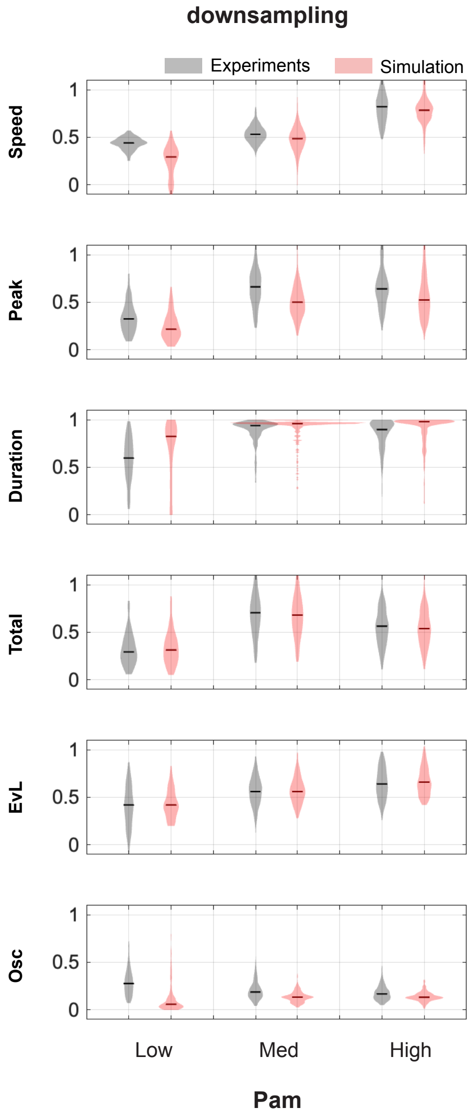

downsampling
Experiments
Simulation
Speed
Peak
Duration
Total
EvL
Osc
0
0.5
1
Low
Med
High
Pam

J

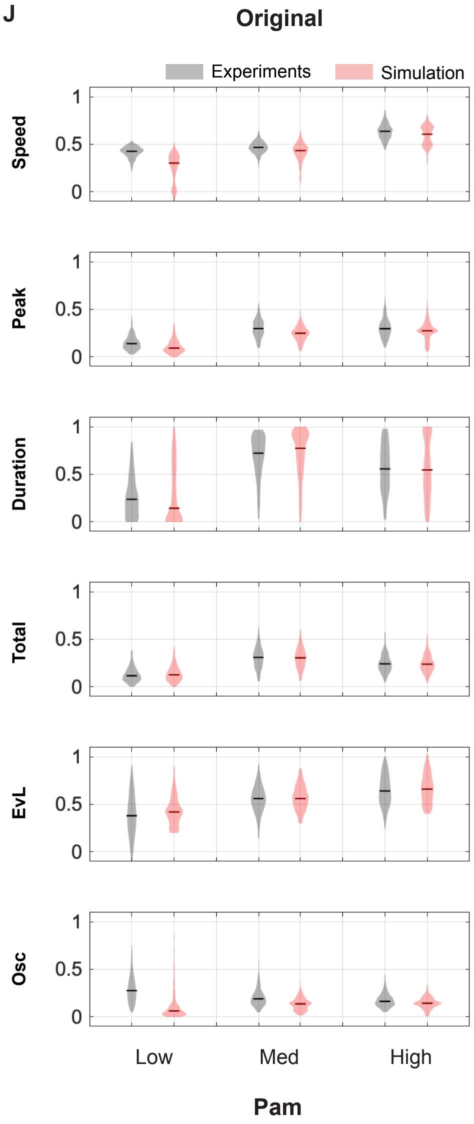

Original
Experiments
Simulation
Speed
Peak
Duration
Total
EvL
Osc
0
0.5
1
Low
Med
High
Pam

K

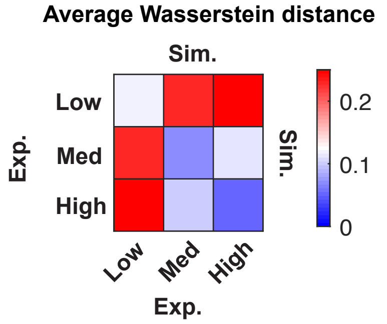

Average Wasserstein distance
Sim.
Low
Med
High
Exp.
0
0.1
0.2

L

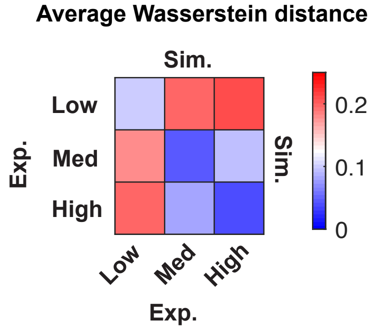

Average Wasserstein distance
Sim.
Low
Med
High
Exp.
0
0.1
0.2

M

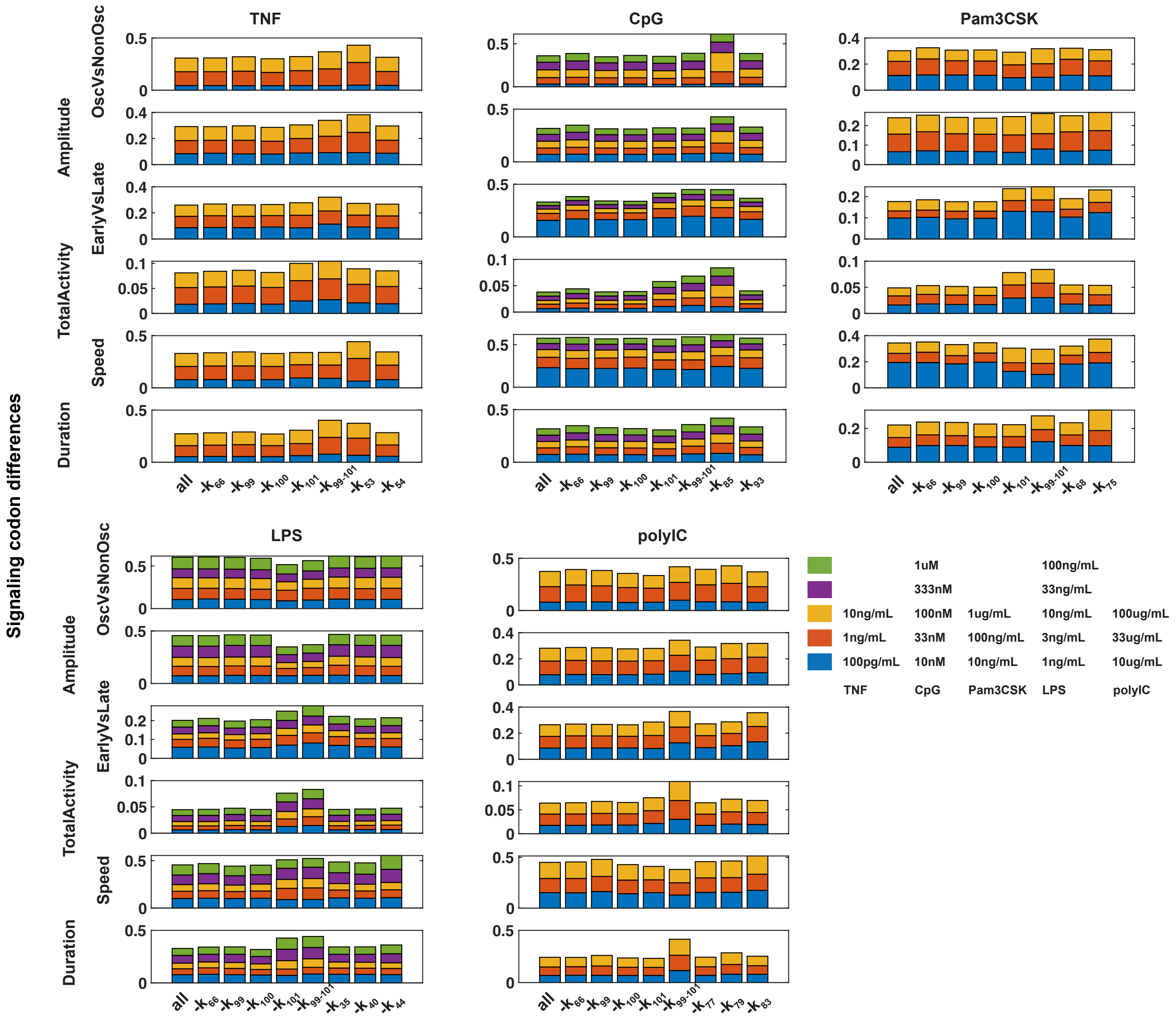

Figure S2. Quantifying the goodness of fit of model simulations to experimental data.

A. (left) Distribution of RMSDs between the experimental and modeling trajectories across different conditions (x-axis). (right) Using trajectory RMSD to determine goodness of fit. Examples of experimental trajectories along with their corresponding fitted trajectories with associated RMSD values indicated. These define the thresholds used in (B).

B. The percentage of successful fits, as defined by the RMSD thresholds defined in (A). Stimulus dose is indicated in the grayscale, L=Low, M=Medium, H=High.

C. Using informative dynamic trajectory features, termed signaling codons to determine the goodness of fit. Violin plots of the distribution of signaling codons in experimental (black) and simulated (red) NFκB trajectories. Each row depicts one of the six signaling codons: activation speed (Speed), maximal amplitude (Peak), duration time (Duration), integral or total activity (Total), front-loaded early vs or late activity (EvL), and oscillatory content (Osc), as detailed on the left. Columns correspond to individual conditions with ligand and dose details on the x-axis labels (L: low; M: medium; H: high dose). The hollow circle in each plot indicates the median of the distribution. The number above each pair of signaling codon distribution is the Wasserstein distance between the corresponding signaling codon distributions; smaller values indicate less discrepancy between simulation and experimental data.

D. Heatmap of Wasserstein distances of signaling codon distributions between simulation and experimental data.

E. The average differences between experimental and simulated signaling codon distribution across the six signaling codons.

F. Confusion matrices illustrating the classification sensitivity of ligand identity information for experimental data (Experiments), and for simulated data calculated and from fitted parameters (Simulation). The

machine learning model (Random Forest) uses NFκB trajectory Signaling Codons as input to encode and predict the ligand information. This is evaluated by five-fold cross-validation.

G. Precision and sensitivity confusion matrix of ligand classification trained and tested across different dataset (specified in the title). The machine learning model (Random Forest) uses NFκB trajectory Signaling Codons as input to predict the ligand classification.

H. Heatmaps showing down-sampled (to half of the original sample size) experimental data (top row) of NFκB signaling in macrophages stimulated with low-, medium-, and high-dose Pam, alongside corresponding simulations (bottom row). Simulations are generated through independent fitting to the down-sampled experimental dataset.

I-J. Violin plots displaying the distribution of signaling codons in NFκB trajectories for experimental (black) and simulated (red) datasets. Panel (I) represents the down-sampled Pam-stimulated dataset, while panel (J) pertains to the original Pam-stimulated dataset.

K-L. Heatmap depicting the average Wasserstein distances between Signaling Codon distributions within and between experiments and simulations across different conditions for down-sampled Pam-stimulated dataset (K) and original Pam-stimulated dataset (L). Diagonal elements (predominantly blue) reflect distances between corresponding experimental and simulated data for each condition. The lower triangular matrix illustrates distances among experimental conditions, while the upper triangular matrix represents distances among simulation conditions. Conditions (Low: low dose; Med: medium dose; High: high dose) are labeled along the x- and y-axes. The intensity of the color corresponds to the magnitude of the average Wasserstein distance.

M. Stacked bar plot illustrating the average signaling codon differences between experimental data and simulations for five ligands (specified in the title) across different doses (color-coded as explained in the bottom-right corner). The analysis is conducted under different parameter fitting sets: "all" represents the control group where all selected parameters are fitted, while "-k_{99}" indicates that all parameters except k_{99} are fitted. Similar nomenclature applies for other parameter sets.

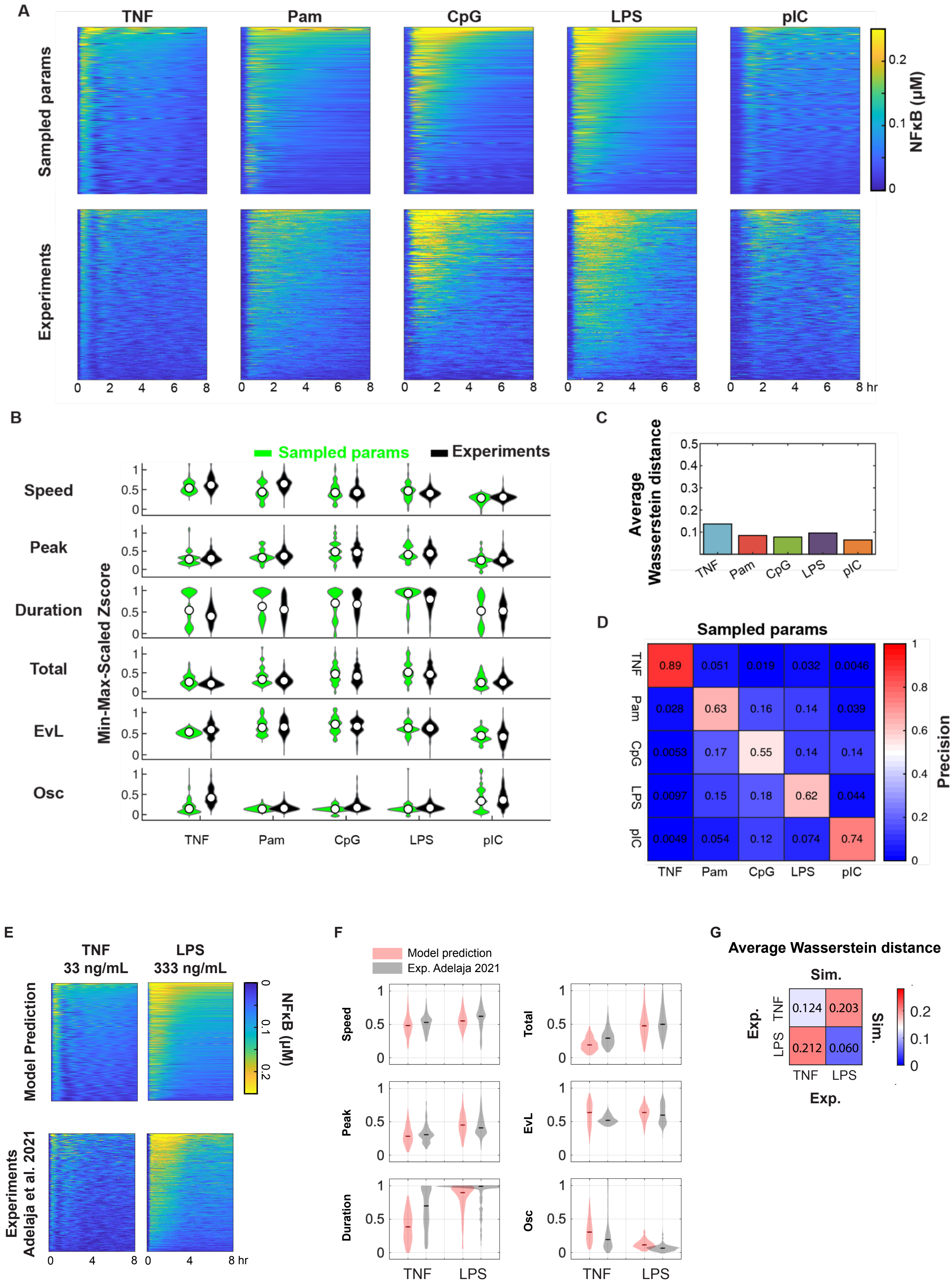

A
TNF
Pam
CpG
LPS
pIC
Sampled params
Experiments
NFκB (μM)
0.2
0.1
0
0 2 4 6 8 hr
B
Sampled params
Experiments
Speed
Peak
Duration
Total
EvL
Osc
Min-Max-Scaled Zscore
TNF Pam CpG LPS pIC
C
Average Wasserstein distance
TNF Pam CpG LPS pIC
D
Sampled params
TNF 0.89 0.051 0.019 0.032 0.0046
Pam 0.028 0.63 0.16 0.14 0.039
CpG 0.0053 0.17 0.55 0.14 0.14
LPS 0.0097 0.15 0.18 0.62 0.044
pIC 0.0049 0.054 0.12 0.074 0.74
Precision
E
TNF 33 ng/mL
LPS 333 ng/mL
Model Prediction
Experiments Adelaja et al. 2021
NFκB (μM)
0 4 8 hr
F
Model prediction
Exp. Adelaja 2021
Speed
Total
Peak
EvL
Duration
Osc
TNF LPS
G
Average Wasserstein distance
Sim.
Exp.
TNF LPS
0.124 0.203
0.212 0.060

H

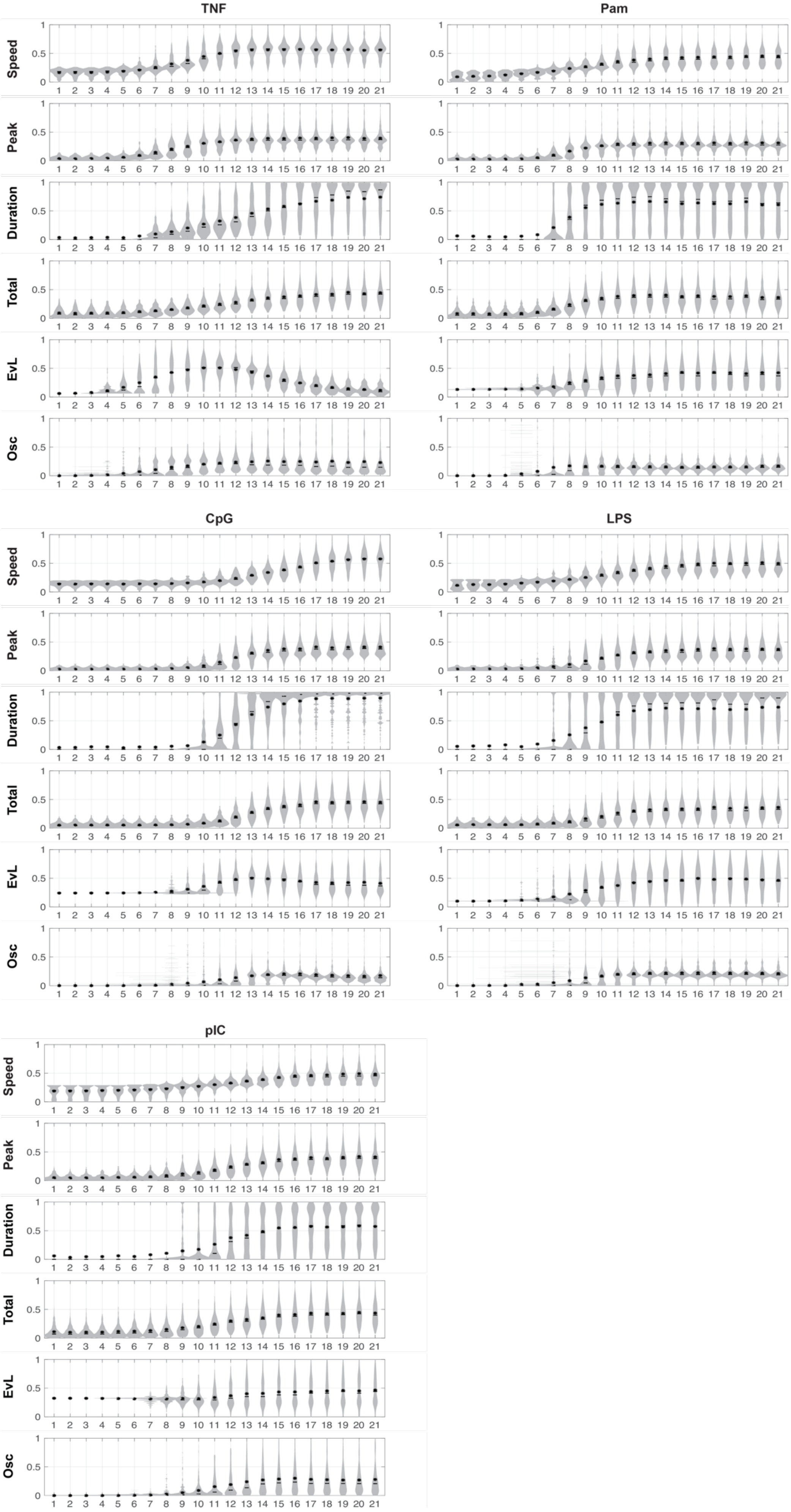
TNF
Pam
CpG
LPS
pIC
Speed
Peak
Duration
Total
EvL
Osc
1 2 3 4 5 6 7 8 9 10 11 12 13 14 15 16 17 18 19 20 21

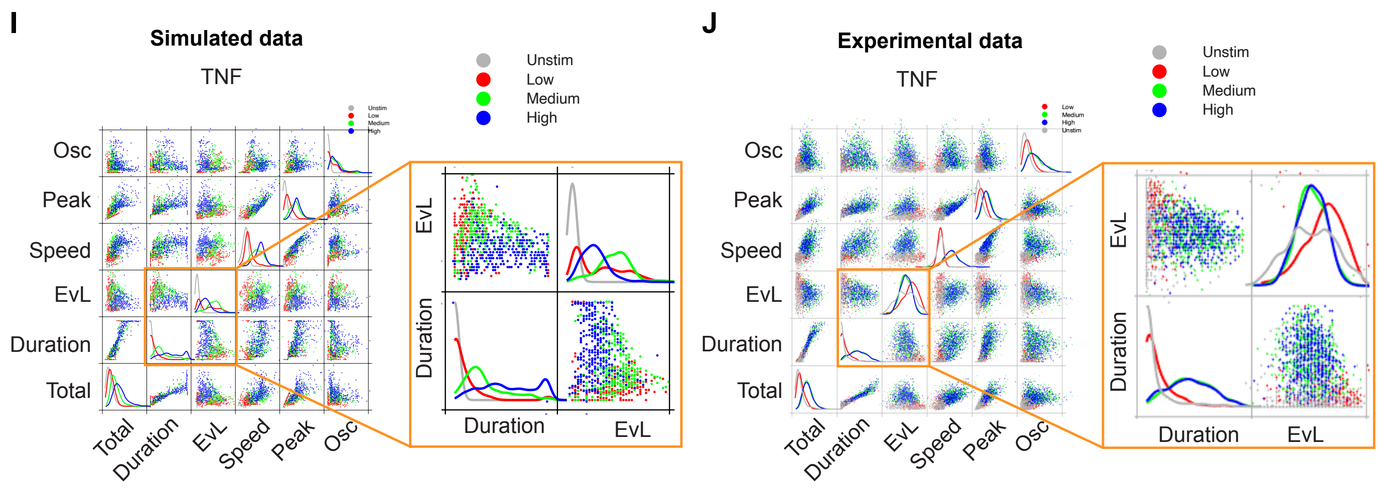

Figure S3. Forward predictions by sampling model parameters from distributions

A. The heatmaps of both experimentally obtained and simulated NFκB signaling trajectories in response to five high-dose ligands (columns: TNF, Pam, CpG, LPS, pIC). The model-generated trajectories used sampled parameters. The y-axis denotes individual cells ordered by Total activity.

B. Violin plots comparing distributions of signaling codons derived from the NFκB trajectories generaledt experimentally (black) or by model simulations with sampled parameters (green) and experimental (red). Each row corresponds to one of the six signaling codons: Speed, Peak, Duration, Total, EvL, and Osc, as detailed on the left. Columns relate to each condition, with high-dose ligand details on the x-axis labels. The hollow circle in each plot indicates the median of the distribution.

C. Average Wasserstein distances between signaling codon distributions from experimental and simulated data.

D. Confusion matrices illustrate the classification precision of ligand identity information for simulated data calculated from sampled parameters (Sampled). The machine learning model (Random forest) uses NFκB trajectory signaling codons as input to predict the ligand information. This is evaluated by five-fold cross-validation. For comparison see Figure 2D for similar confusion matrices for experimental or model fitted trajectories.

E. Heatmaps showing model-predicted NFκB signaling trajectories in response to 33 ng/mL TNF and 333 ng/mL LPS, both outside the training range. Corresponding experimentally measured trajectories (data [1]) are included for validation. The y-axis represents individual cells, ordered by Total activity.

F. Violin plots comparing the distributions of signaling codons derived from model-predicted (red) and experimentally observed (black) NFκB trajectories [1], corresponding to Figure S3G. Each row represents one of six signaling codons: Speed, Peak, Duration, Total, EvL, and Osc, as indicated on the y-axis. The x-axis specifies the TNF or LPS condition. Short horizontal lines within the plots indicate the median of each distribution.

G. Heatmap illustrating average Wasserstein distances between signaling codon distributions within and between experimental and simulated datasets for TNF and LPS stimulation, corresponding to Figure S3G.

H. Violin plots of signaling codons (labeled on left) distribution of NFκB signaling trajectories in response to different doses (dose index specified in x-axis) of five ligands (labeled in the title). '*' represents the mean value, '-' represents the median value.

I-J. Scatter plots of the 2D signaling codon distribution of TNF-response experimental (I) and simulated (J) trajectories. The diagonal shows distributions of the corresponding signaling codon specified on x- and y-axes. Off diagonal are the scatter plots of two signaling codon distributions specified. Each point represents the signaling codon value corresponding to a single trajectory. Colors indicate the stimulus dose. Insets are zoomed-in version for EvL and Duration codon distribution.

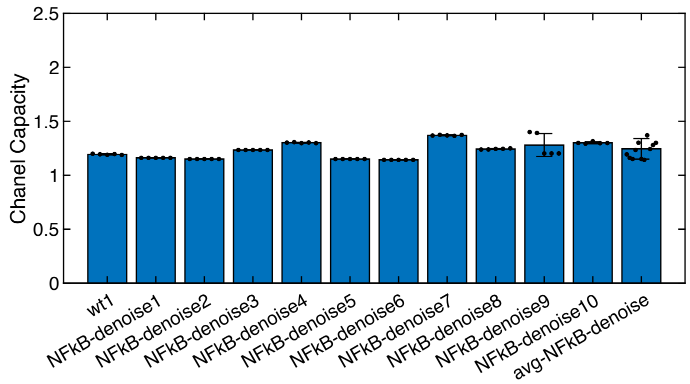

Figure S4. The core module does not limit channel capacity. (Related to Figure 4)
Robustness analysis for different denoising strategies. Channel capacity of the network with noise present in all three modules (all noise), compared to denoising in the core NFκB-IκBα module (core) using different parameter values within the core module. Denoise1 represents the parameter value of a representative cell, while denoise 2-10 corresponds to nine other randomly selected sets of parameter values. Denoising implies that all sampled virtual cells adopt the same parameter values for the respective module, in this case, the core module. MI is calculated five times per condition, displayed as individual data points. The final bar (avg-NFκB-denoise) shows the average channel capacity across all denoising strategies tested, with individual data reflecting the values of the other bars (i.e., the average MI for different conditions). Data are presented as mean ± SD.

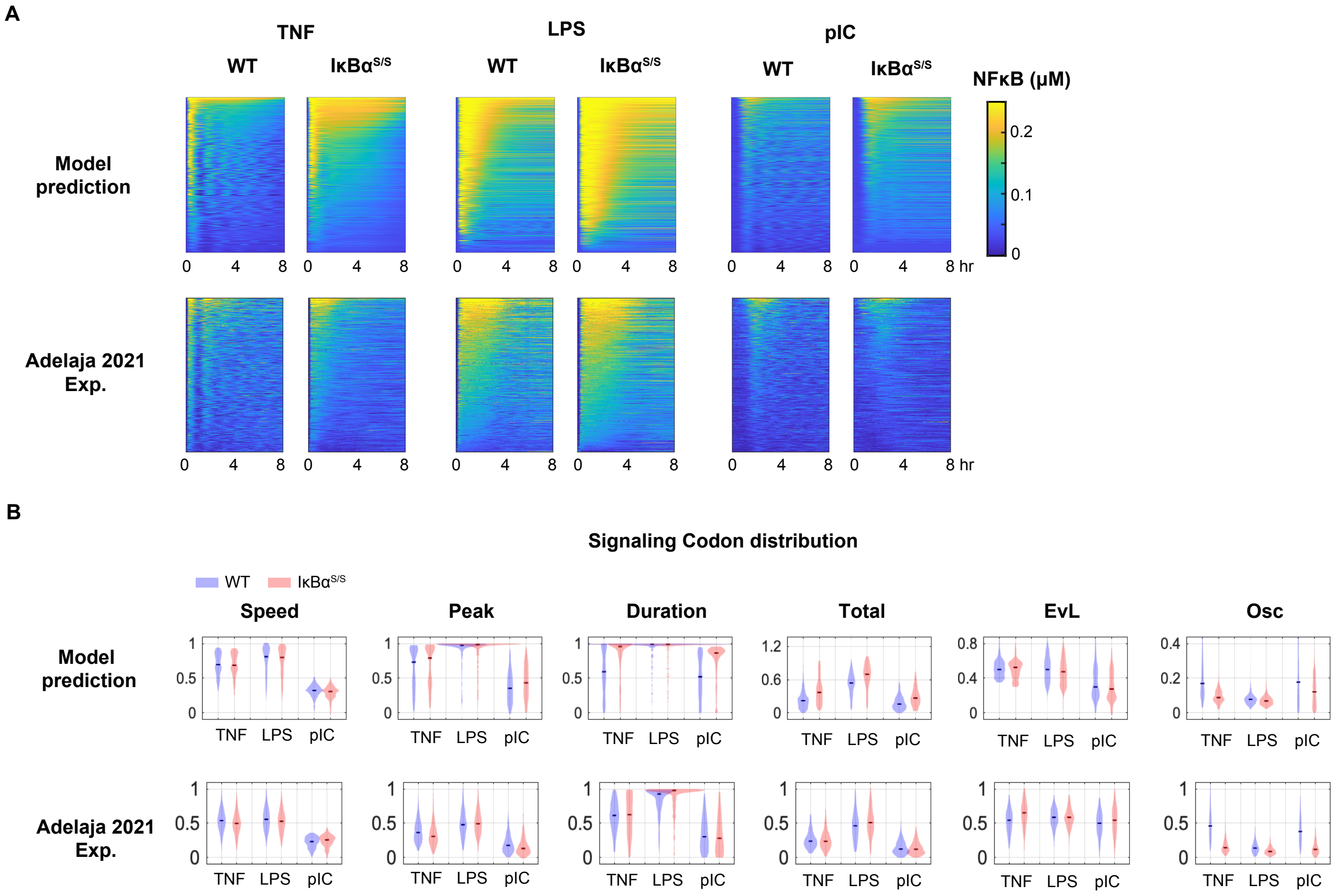

Figure S5. Model predictions on the IκBα$^{S/S}$ promoter mutant. (Related to Figure 5)

A. Heatmaps of simulated (top row) and experimental measured (bottom row, data from [1]) NFκB signaling trajectories in macrophages stimulated by three ligands (TNF left two columns, LPS middle two, pIC right two) for wild type (WT, left panels) and the IκBα$^{S/S}$ promoter mutant (right panels). In each subpanel, the y-axis denotes individual cells, the x-axis represents time, and color intensity indicates NFκB abundance in accordance with the color bar.

B. Violin plots of signaling codon (min-max rescaled z-score) distributions derived from the simulated (top row) and experimental measured (bottom row, data from [1]) NFκB trajectories for wild type (WT, blue distributions) and IκBα$^{S/S}$ promoter mutant (red distributions). Each column corresponds to one of the six signaling codons: Total, Duration, EvL, Speed, Peak, and Osc, as detailed on the subtitles of each subpanel. Within each subpanel, six violin plots correspond to three conditions (with two genotype for each condition), with ligand details on the x-axis labels. '-' represents the median value.

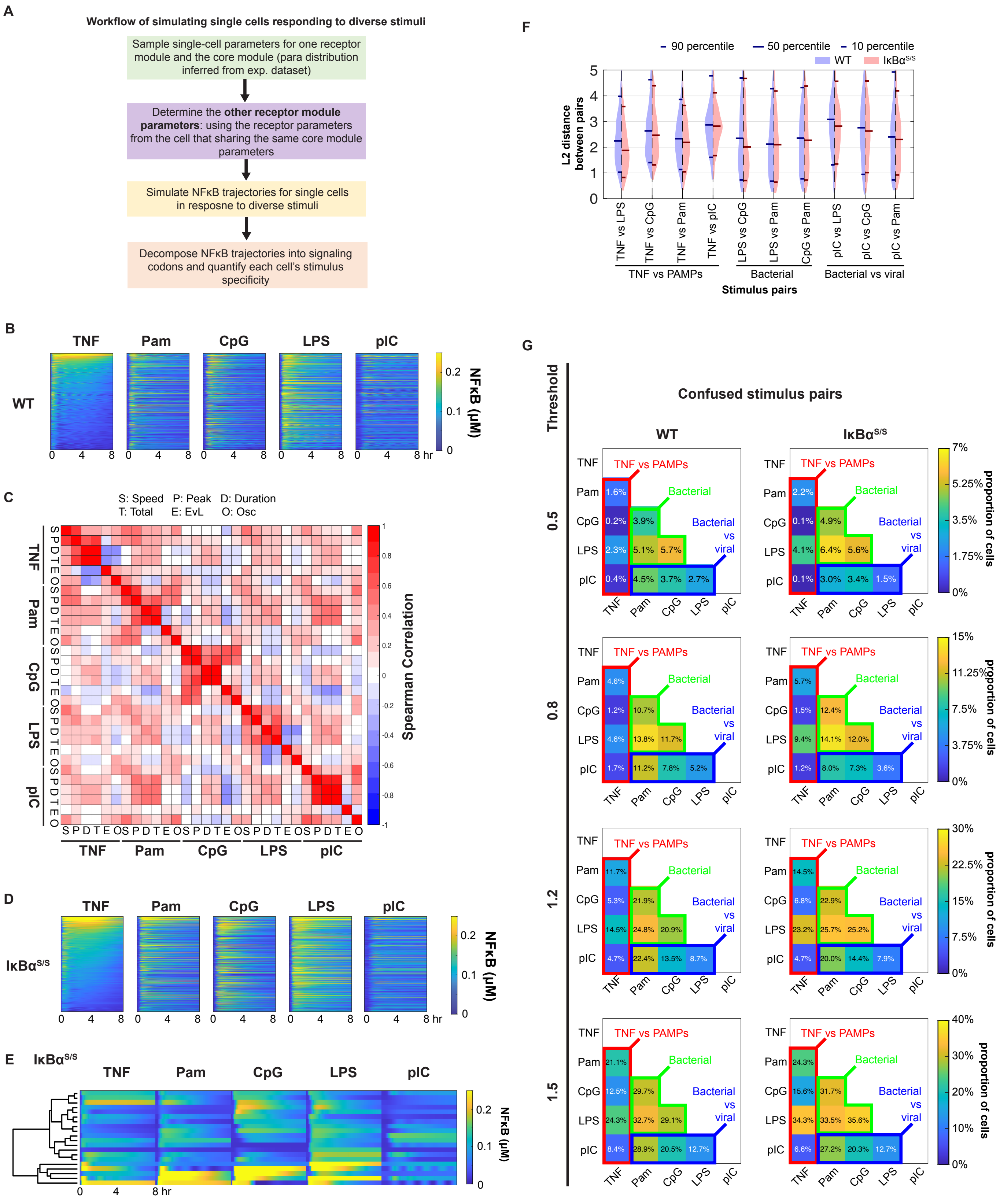

Figure S6. NFκB responses by individual NFκB signaling network to five single-ligand stimuli. (Related to Figure 6)

A. Workflow of generating heterogenous simulations of single cells response to diverse stimuli.
B. Heatmaps of simulated NFκB signaling trajectories by individual NFκB signaling networks in response to 5 ligands, sorted by Total of TNF stimulation.
C. Spearman Correlation among 30 signaling codons associated with each individual NFκB signaling network —five stimuli by six signaling codons.
D. Heatmaps of the simulated NFκB signaling trajectories of individual NFκB signaling networks responding to 5 ligands in the IκBα$^{S/S}$ promoter mutant sorted by Total of TNF stimulation.

E. Heatmaps of NFκB signaling trajectories by 20 individual IκBα$^{S/S}$ NFκB signaling networks responding to 5 ligands, sorted by hierarchical clustering of 5 ligand stimulation trajectories.
F. Violin plots depicting the distribution of l2 distance in the signaling codon space between stimulus pairs (specified in the x-axis), for WT and IκBα$^{S/S}$. 10th, 50th, and 90th percentile are marked from bottom to top within each distribution violin plot.
G. Heatmap of the proportion of individual NFκB signaling networks that confuse the indicated stimulus pairs (specified on x-axis and y-axis) using different thresholds (specified on the left) for WT and IκBα$^{S/S.}$

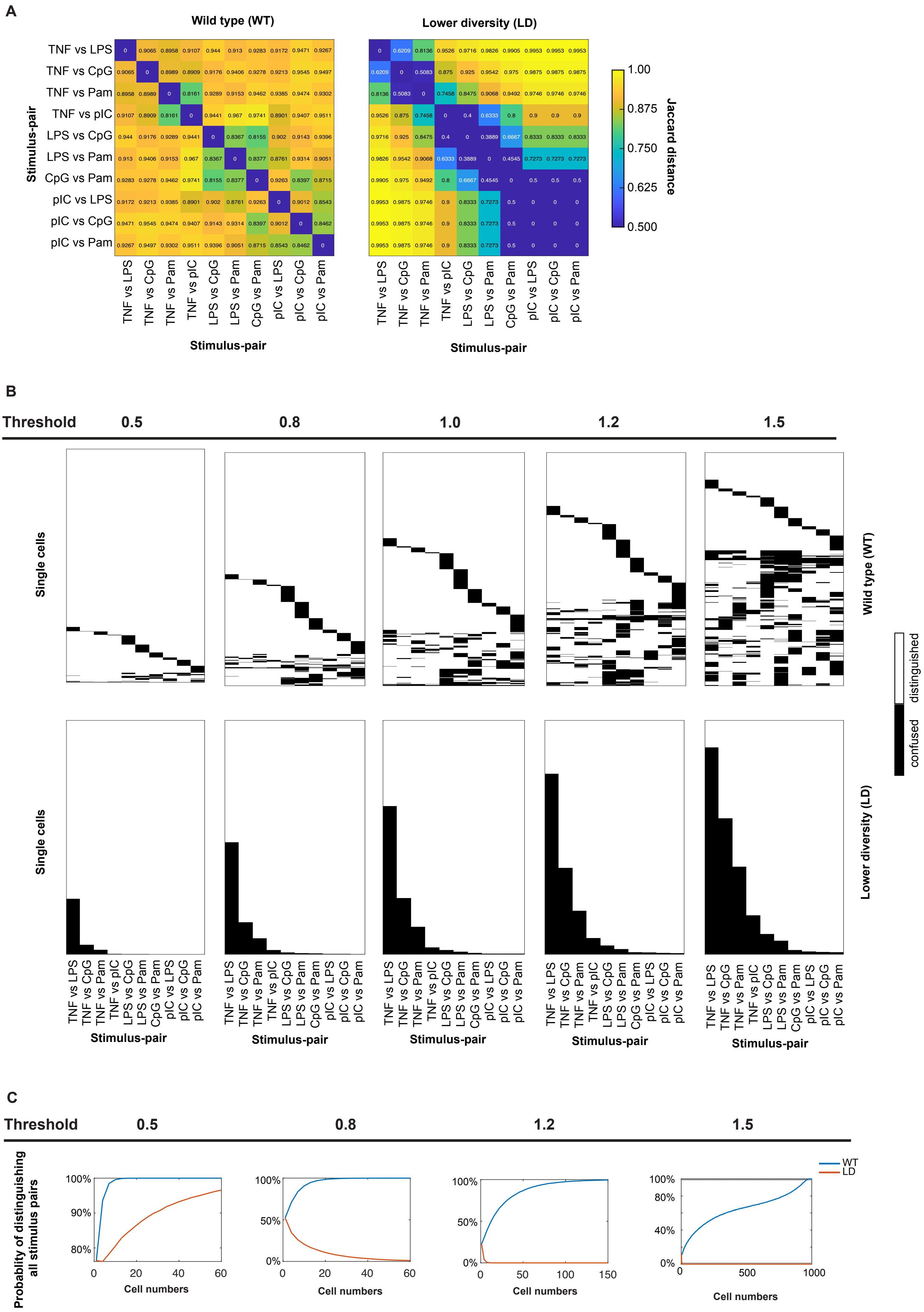
A
Wild type (WT)
Lower diversity (LD)
Stimulus-pair
TNF vs LPS
TNF vs CpG
TNF vs Pam
TNF vs pIC
LPS vs CpG
LPS vs Pam
CpG vs Pam
pIC vs LPS
pIC vs CpG
pIC vs Pam
Jaccard distance
1.00
0.875
0.750
0.625
0.500
B
Threshold
0.5
0.8
1.0
1.2
1.5
Single cells
Wild type (WT)
Lower diversity (LD)
distinguished
confused
C
Threshold
0.5
0.8
1.2
1.5
Probability of distinguishing all stimulus pairs
Cell numbers
WT
LD

Figure S7. Quantifying the heterogeneity of stimulus-pair confusion in scSRS. (Related to Figure 7)

A. Heatmap of the Jaccard distances of instances of stimulus-pair confusion (specified on x-axis and y-axis) for the stimulus-distinction threshold of 1.0. Data for wild-type (WT, left panel) and lower diversity (LD) cell populations are shown (corresponding to Figure 7A-B).

B. Binary category map indicating confused stimulus pairs (specified on x-axis) of single-cell NFκB signaling networks (virtual cells) using different stimulus-distinction thresholds (specified on the top) for WT (top row) and LD (bottom row) cell populations.

C. Line graph of the probability of distinguishing all 10 stimulus pairs (defined by at least two-thirds of the cells in the consortium distinguishing each pair) as a function of the number of macrophages in a consortium, using different stimulus-distinction thresholds (specified on the top), comparing cell populations in which the stimulus-pair confusion pattern has lower diversity (LD) than wild-type (WT).

Part II: Supplementary Notes for Methods
Modeling single-cell heterogeneity in signaling dynamics of macrophages reveals principles of information transmission

Xiaolu Guo[1,2], Adewunmi Adelaja[1,2,#], Apeksha Singh[1,2], Roy Wollman[1,3] Alexander Hoffmann[*,1,2]

May 2024

1 Department of Microbiology, Immunology, and Molecular Genetics (MIMG), University of California Los Angeles, Los Angeles, USA
2 Institute for Quantitative and Computational Biosciences, University of California Los Angeles, Los Angeles, USA
3 Department of Integrative Biology and Physiology, University of California Los Angeles, Los Angeles, USA
# Current address: Harvard combined Dermatology Residency Training Program, Boston, MA, USA
***** Corresponding author: ahoffmann@ucla.edu

Contents

1 Experimental information: single-ligand stimulation 3
1.1 Macrophage Cell Culture . . . 3
1.2 Macrophage stimulation conditions . . . 3
1.3 Live-cell imaging . . . 3
1.4 Image analysis and processing . . . 3

2 NFκB system equations 3
2.1 TLR4 module (LPS) . . . 4
2.2 TNFR1 module (TNF) . . . 5
2.3 Pam3CSK module (P3CSK) . . . 5
2.4 TLR3 module (polyIC) . . . 5
2.5 TLR9 module (CpG) . . . 6
2.6 Adaptor module . . . 6
2.7 Core Module . . . 7
2.8 Time delay (Core module) . . . 8
2.9 Competition between CpG and polyIC . . . 8
2.10 notations . . . 9

3 Scaling factor for NFκB standard units to arbitrary units 9

4 Mathematical formulation of signaling codons 10

5 Statistical Model 12
5.1 Parameter selection . . . 13
5.2 Staistical model . . . 13
5.3 Algorithms for MLE and MAP . . . 14

6 Generate simulation data 17
6.1 Single ligand stimulation data . . . 17
6.2 Dual-ligand stimulation data . . . 18
6.3 Multi-ligand stimulation data . . . 19
6.4 Single-cell data responding to different ligands . . . 20
6.5 Test different matching methods . . . 20

7 Complex relationships between biochemical parameters and signaling codons 21

1 Experimental information: single-ligand stimulation

1.1 Macrophage Cell Culture

Bone marrow-derived macrophages (BMDMs) were prepared by culturing bone marrow monocytes from femurs of 8-12 week old mice expressing mVenus-RelA from the endogenous RelA locus [1] in CMG 14-12-conditioned medium or L929-conditioned medium using standard methods [6, 19]. BMDMs were re-plated in imaging dishes on day 4, media exchanged with fresh media on day 6, then BMDMs were stimulated on day 7.

1.2 Macrophage stimulation conditions

BMDMs were stimulated with indicated concentrations of lipopolysaccharide (LPS, Sigma Aldrich), murine TNF (R&D), a TLR1/2 agonist, the synthetic triacylated lipoprotein Pam3CSK4 (P3C4), a TLR3 agonist, low molecular weight polyinosine-polycytidylic acid (Poly(I:C) (p(I:C)), Invivogen), a TLR9 agonist, the synthetic CpG ODN 1668 (CpG) (Invivogen). Stimulus conditions LPS, Pam3CSK4, Poly(I:C), and CpG were co-applied with TNF inhibitor (soluble TNFR2) in conditioned culture medium.

1.3 Live-cell imaging

Bone-marrow macrophages were replated on day 4 at 20,000 or $15,000/cm^2$ in an 8-well ibidi SlideTek chamber, for imaging at an appropriate density (approx. 60,000/cm2) on day 6 or day 7. 2 hours prior to stimulation, a solution of 2.5 ng/mL Hoechst 33342 was added to the BMDM culture media. After the start of imaging, additional culture media containing stimulus (TNF, LPS, poly(I:C), CpG, or P3C4) was injected into the chamber in situ. Cells were imaged at 5-minute intervals on a Zeiss Axio Observer platform with live-cell incubation, using epifluorescent excitation from a Sutter Lambda XL light source. Images were recorded on a Hamamatsu Orca Flash 2.0 CCD camera.

1.4 Image analysis and processing

Microscopy time-lapse images were exported for single-cell tracking and measurement in MATLAB R2016a. The tracking routines followed those used in earlier work [16]. Briefly, cells were identified using DIC images, then segmented, guided by markers from the Hoechst image. Segmented cells were linked into trajectories across successive images, then nuclear and cytoplasmic boundaries were saved and used to define measurement regions in other fluorescent channels, including mVenus-NFκB. Nuclear NFκB levels were quantified on a per-cell basis, normalized to image background levels, then were baseline-subtracted. Mitotic cells, as well as cells that drifted out of the field of view, were excluded from analysis. The toolboxes used for this analysis are available at GitHub (`https://github.com/Adewunmi91/MACKtrack`).

2 NFκB system equations

The ordinary differential equation model of the NFκB signaling network aims to predict NFκB signaling dynamics in response to five pro-inflammatory ligands: Tumor Necrosis Factor (TNF) and PAMPs, namely the TLR2 ligand Pam3CysSerLys4 (Pam), the TLR9 ligand Cytosine-phosphate-Guanine (CpG), the TLR4 ligand Lipopolysaccharide (LPS), and the TLR3 ligand Polyinosinic:polycytidylic acid (pIC), at several doses (Figure 1A). The published model simulation produces an NFκB trajectory for each ligand that is representative of the observed data [1].

The mechanistic model contains 52 species (Table S1), 101 reactions, and 133 parameters (Table S2, Eqs (1) to (52)). It is organized into five receptor modules: the LPS module (reactions 27 to 46 in Table

S2), the TNF module (reactions 53 to 65 in Table S2), the Pam module (reactions 68 to 76 in Table S2), the pIC module (reactions 40 to 43 in Table S2), and the CpG module (reactions 85 to 94 in Table S2) (Figure 1A in main manuscript). These receptor modules respond to five inflammatory ligands and feed into a common core module (reactions 1 to 26, 47 to 52, 66 to 67 in Table S2) that includes MyD88 module, TAK activation module, and the core module of NFκB-IKK-IκBα feedback loop. The node kinase IKK activated by TAK1 and regulates the NFκB-IκBα negative feedback loop. These equations are grouped into five receptor modules,

The model is adapted from Adelaja et al. [1], which is based on prior formulations of TNFR and TLR4 signaling models [6, 22], IKK deactivation and reactivation kinetics [4], and other relevant mechanisms. Molecular biochemical reaction parameters in the model are derived from previous experimental studies, such as the oscillatory dynamics of NFκB signaling in response to TNF [3, 9, 11] and IKK activity levels in response to LPS or TNF [3, 13, 17, 20, 22, 23]. The model also considers TLR1/2, TLR3, and TLR9 receptor abundance [15], among other factors.

The NFκB signaling network can be modeled by ODEs [1]. For feasibility of parameter estimation algorithm, we replaced the time delay for NFκB regulated IκBα transcription with cascade reactions (49)(50)(51)(52). The following sections list the details of the models.

2.1 TLR4 module (LPS)

$$\frac{\mathrm{d}}{\mathrm{d}t}LPS = -(\psi_{27,1} * LPS * CD14 * \psi_{27,2} + (\psi_{28,1} * CD14LPS * \psi_{28,2}) \tag{1}$$

$$\begin{aligned}\frac{\mathrm{d}}{\mathrm{d}t}CD14 = &- \psi_{27,1} * LPS * CD14 - \psi_{30,1} * CD14 \\ &- \psi_{70,1} * Pam3CSK * CD14 + \psi_{28,1} * CD14LPS + \psi_{29,1} + \psi_{71,1} * CD14_{P3CSK}\end{aligned} \tag{2}$$

$$\begin{aligned}\frac{\mathrm{d}}{\mathrm{d}t}CD14LPS = &- \psi_{28,1} * CD14LPS - \psi_{31,1} * CD14LPS * TLR4 \\ &- \psi_{36,1} * CD14LPS + \psi_{27,1} * LPS * CD14 + \psi_{32,1} * TLR4LPS \\ &+ \psi_{37,1} * CD14LPSen\end{aligned} \tag{3}$$

$$\begin{aligned}\frac{\mathrm{d}}{\mathrm{d}t}CD14LPSen = &- \psi_{33,1} * CD14LPSen * TLR4en - \psi_{37,1} * CD14LPSen \\ &- \psi_{42,1} * CD14LPSen + \psi_{34,1} * TLR4LPSen + \psi_{36,1} * CD14LPS\end{aligned} \tag{4}$$

$$\begin{aligned}\frac{\mathrm{d}}{\mathrm{d}t}TLR4 = &- \psi_{31,1} * CD14LPS * TLR4 \\ &- \psi_{38,1} * TLR4 + \psi_{32,1} * TLR4LPS + \psi_{35,1} + \psi_{39,1} * TLR4en\end{aligned} \tag{5}$$

$$\begin{aligned}\frac{\mathrm{d}}{\mathrm{d}t}TLR4en = &- \psi_{33,1} * CD14LPSen * TLR4en - \psi_{39,1} * TLR4en \\ &- \psi_{43,1} * TLR4en + \psi_{34,1} * TLR4LPSen + \psi_{38,1} * TLR4\end{aligned} \tag{6}$$

$$\begin{aligned}\frac{\mathrm{d}}{\mathrm{d}t}TLR4LPS = &- \psi_{32,1} * TLR4LPS - \psi_{40,1} * TLR4LPS \\ &+ \psi_{31,1} * CD14LPS * TLR4 + \psi_{41,1} * TLR4LPSen\end{aligned} \tag{7}$$

$$\begin{aligned}\frac{\mathrm{d}}{\mathrm{d}t}TLR4LPSen = &- \psi_{34,1} * TLR4LPSen - \psi_{41,1} * TLR4LPSen \\ &- \psi_{44,1} * TLR4LPSen + \psi_{33,1} * CD14LPSen * TLR4en + \psi_{40,1} * TLR4LPS\end{aligned} \tag{8}$$

2.2 TNFR1 module (TNF)

$$\frac{\mathrm{d}}{\mathrm{d}t}TNF = -\,\psi_{53,1} * TNF - (\psi_{56,1} * TNF * TNFR * \psi_{56,2}) + (\psi_{57,1} * TNFR_{TNF,1} * \psi_{57,2}) \tag{9}$$

$$\frac{\mathrm{d}}{\mathrm{d}t}TNFR = -\,\psi_{55,1} * TNFR - \psi_{56,1} * TNF * TNFR + \psi_{54,1} + \psi_{57,1} * TNFR_{TNF} \tag{10}$$

$$\begin{aligned}\frac{\mathrm{d}}{\mathrm{d}t}TNFR_{TNF} = &-\,\psi_{57,1} * TNFR_{TNF} - \psi_{58,1} * TNFR_{TNF} \\ &- \psi_{59,1} * TNFR_{TNF,1} * TTR + \psi_{56,1} * TNF * TNFR + \psi_{60,1} * C1_{off}\end{aligned} \tag{11}$$

$$\frac{\mathrm{d}}{\mathrm{d}t}TTR = -\,\psi_{59,1} * TNFR_{TNF,1} * TTR + \psi_{60,1} * C1_{off} \tag{12}$$

$$\begin{aligned}\frac{\mathrm{d}}{\mathrm{d}t}C1_{off} = &-\,\psi_{60,1} * C1_{off} - \psi_{61,1} * C1_{off} - \psi_{62,1} * C1_{off} + \psi_{59,1} * TNFR_{TNF,1} * TTR \\ &+ \psi_{63,1} * C1\end{aligned} \tag{13}$$

$$\frac{\mathrm{d}}{\mathrm{d}t}C1 = -\,\psi_{63,1} * C1 - \psi_{64,1} * C1 + \psi_{62,1} * C1_{off} \tag{14}$$

2.3 Pam3CSK module (P3CSK)

$$\frac{\mathrm{d}}{\mathrm{d}t}Pam3CSK = -\,(\psi_{70,1} * Pam3CSK * CD14 * \psi_{70,2}) + (\psi_{71,1} * CD14_{P3CSK,1} * \psi_{71,2}) \tag{15}$$

$$\frac{\mathrm{d}}{\mathrm{d}t}TLR2 = -\,\psi_{69,1} * TLR2 - \psi_{72,1} * CD14_{P3CSK,1} * TLR2 + \psi_{68,1} + \psi_{74,1} * TLR2_{P3CSK} \tag{16}$$

$$\begin{aligned}\frac{\mathrm{d}}{\mathrm{d}t}CD14_{P3CSK} = &-\,\psi_{71,1} * CD14_{P3CSK} - \psi_{72,1} * CD14_{P3CSK,1} * TLR2 - \psi_{73,1} * CD14_{P3CSK} \\ &+ \psi_{70,1} * Pam3CSK * CD14 + \psi_{74,1} * TLR2_{P3CSK}\end{aligned} \tag{17}$$

$$\frac{\mathrm{d}}{\mathrm{d}t}TLR2_{P3CSK} = -\,\psi_{74,1} * TLR2_{P3CSK} - \psi_{75,1} * TLR2_{P3CSK} + \psi_{72,1} * CD14_{P3CSK,1} * TLR2 \tag{18}$$

2.4 TLR3 module (polyIC)

$$\frac{\mathrm{d}}{\mathrm{d}t}polyIC = -\,(\psi_{79,1} * (polyIC^{\psi_{79,3}})/((polyIC^{\psi_{79,3}}) + (\psi_{79,2}^{\psi_{79,3}})) * \psi_{79,4}) + (\psi_{80,1} * polyIC_{en,1} * \psi_{80,2}) \tag{19}$$

$$\begin{aligned}\frac{\mathrm{d}}{\mathrm{d}t}polyIC_{en} = &-\,\psi_{80,1} * polyIC_{en} - \psi_{81,1} * polyIC_{en,1} * TLR3 + \psi_{79,1} * (polyIC^{\psi_{79,3}})/((polyIC^{\psi_{79,3}}) \\ &+ (\psi_{79,2}^{\psi_{79,3}})) + \psi_{82,1} * TLR3_{polyIC}\end{aligned} \tag{20}$$

$$\frac{\mathrm{d}}{\mathrm{d}t}TLR3 = -\,\psi_{78,1} * TLR3 - \psi_{81,1} * polyIC_{en,1} * TLR3 + \psi_{77,1} + \psi_{82,1} * TLR3_{polyIC} \tag{21}$$

$$\frac{\mathrm{d}}{\mathrm{d}t}TLR3_{polyIC} = -\,\psi_{82,1} * TLR3_{polyIC} - \psi_{83,1} * TLR3_{polyIC} + \psi_{81,1} * polyIC_{en,1} * TLR3 \tag{22}$$

2.5 TLR9 module (CpG)

$$\frac{\mathrm{d}}{\mathrm{d}t}CpG = -(\psi_{88,1} * (CpG^{\psi_{88,3}})/((CpG^{\psi_{88,3}}) + (\psi_{88,2}^{\psi_{88,3}})) * \psi_{88,4}) + (\psi_{89,1} * CpG_{en,1} * \psi_{89,2}) \tag{23}$$

$$\frac{\mathrm{d}}{\mathrm{d}t}CpG_{en} = -\psi_{89,1} * CpG_{en} - \psi_{90,1} * CpG_{en,1} * TLR9 + \psi_{88,1} * (CpG^{\psi_{88,3}})/((CpG^{\psi_{88,3}}) + (\psi_{88,2}^{\psi_{88,3}})) + \psi_{91,1} * TLR9_{CpG} \tag{24}$$

$$\frac{\mathrm{d}}{\mathrm{d}t}TLR9 = -\psi_{86,1} * TLR9 - \psi_{90,1} * CpG_{en,1} * TLR9 + \psi_{85,1} + \psi_{91,1} * TLR9_{CpG} \tag{25}$$

$$\frac{\mathrm{d}}{\mathrm{d}t}TLR9_{CpG} = -\psi_{91,1} * TLR9_{CpG} - \psi_{92,1} * TLR9_{CpG,1} * TLR9_{N} - \psi_{93,1} * TLR9_{CpG} + \psi_{90,1} * CpG_{en,1} * TLR9 \tag{26}$$

$$\frac{\mathrm{d}}{\mathrm{d}t}TLR9_{N} = -\psi_{87,1} * TLR9_{N} - \psi_{92,1} * TLR9_{CpG,1} * TLR9_{N} + \psi_{85,1} \tag{27}$$

2.6 Adaptor module

$$\begin{aligned}\frac{\mathrm{d}}{\mathrm{d}t}MyD88_{off} = &-\frac{\psi_{45,1} * (TLR4LPS^{\psi_{45,2}})}{((TLR4LPS^{\psi_{45,2}}) + (\psi_{45,3}^{\psi_{45,2}}))} * MyD88_{off} \\ &-\frac{\psi_{76,1} * (TLR2P3CSK^{\psi_{76,2}})}{((TLR2P3CSK^{\psi_{76,2}}) + (\psi_{76,3}^{\psi_{76,2}}))} * MyD88_{off} \\ &-\frac{\psi_{94,1} * (TLR9CpG^{\psi_{94,2}})}{(TLR9CpG^{\psi_{94,2}}) + (\psi_{94,3}^{\psi_{94,2}}))} * MyD88_{off} + \psi_{46,1} * MyD88\end{aligned} \tag{28}$$

$$\begin{aligned}\frac{\mathrm{d}}{\mathrm{d}t}MyD88 = &-\psi_{46,1} * MyD88 + \psi_{45,1} * \frac{(TLR4LPS^{\psi_{45,2}})}{((TLR4LPS^{\psi_{45,2}}) + (\psi_{45,3}^{\psi_{45,2}})) * MyD88_{off}} \\ &+\frac{\psi_{76,1} * (TLR2P3CSK^{\psi_{76,2}})}{((TLR2P3CSK^{\psi_{76,2}}) + (\psi_{76,3}^{\psi_{76,2}})) * MyD88_{off}} \\ &+\frac{\psi_{94,1} * (TLR9CpG^{\psi_{94,2}})}{((TLR9CpG^{\psi_{94,2}}) + (\psi_{94,3}^{\psi_{94,2}})) * MyD88_{off}}\end{aligned} \tag{29}$$

$$\frac{\mathrm{d}}{\mathrm{d}t}TRIF_{off} = -\psi_{47,1} * TRIF_{off,1} * TLR4LPSen - \psi_{84,1} * TRIF_{off,1} * TLR3_{polyIC} + \psi_{48,1} * TRIF \tag{30}$$

$$\frac{\mathrm{d}}{\mathrm{d}t}TRIF = -\psi_{48,1} * TRIF + \psi_{47,1} * TRIF_{off,1} * TLR4LPSen + \psi_{84,1} * TRIF_{off,1} * TLR3_{polyIC} \tag{31}$$

$$\frac{\mathrm{d}}{\mathrm{d}t}TRAF6_{off} = -\psi_{49,1} * MyD88 * TRAF6_{off} - \psi_{50,1} * TRIF * TRAF6_{off} + \psi_{51,1} * TRAF6 \tag{32}$$

$$\frac{\mathrm{d}}{\mathrm{d}t}TRAF6 = -\psi_{51,1} * TRAF6 + \psi_{49,1} * MyD88 * TRAF6_{off} + \psi_{50,1} * TRIF * TRAF6_{off} \tag{33}$$

$$\begin{aligned}\frac{\mathrm{d}}{\mathrm{d}t}TAK1_{off} = & -\psi_{52,1} * TRAF6 * TAK1_{off} * \psi_{52,2} \\ & -\psi_{65,1} * C1 * TAK1_{off} * \psi_{52,2} + \psi_{66,1} * TAK1\end{aligned} \tag{34}$$

$$\begin{aligned}\frac{\mathrm{d}}{\mathrm{d}t}TAK1 = & -\psi_{66,1} * TAK1 + \psi_{52,1} * TRAF6 * TAK1_{off} * \psi_{52,2} \\ & + \psi_{65,1} * C1 * TAK1_{off} * \psi_{52,2}\end{aligned} \tag{35}$$

2.7 Core Module

$$\frac{\mathrm{d}}{\mathrm{d}t}stim = 0 \tag{36}$$

$$\begin{aligned}\frac{\mathrm{d}}{\mathrm{d}t}IkBa = & -\psi_{9,1} * IkBa - \psi_{15,1} * IkBa - \psi_{17,1} * IkBa * NFkB \\ & -\psi_{22,1} * IKK * IkBa + \psi_{8,1} * IkBat_{cas2} + (\psi_{11,1} * IkBan * \psi_{11,2}) \\ & + \psi_{19,1} * IkBaNFkB + \psi_{24,1} * IKKIkBa\end{aligned} \tag{37}$$

$$\begin{aligned}\frac{\mathrm{d}}{\mathrm{d}t}IkBan = & -\psi_{11,1} * IkBan - \psi_{16,1} * IkBan - \psi_{18,1} * IkBan * NFkBn \\ & + (\psi_{9,1} * IkBa * \psi_{9,2}) + \psi_{20,1} * IkBaNFkBn\end{aligned} \tag{38}$$

$$\begin{aligned}\frac{\mathrm{d}}{\mathrm{d}t}IkBaNFkB = & -\psi_{13,1} * IkBaNFkB - \psi_{19,1} * IkBaNFkB - \psi_{21,1} * IKK * IkBaNFkB \\ & + (\psi_{14,1} * IkBaNFkBn * \psi_{14,2}) + \psi_{17,1} * IkBa * NFkB + \psi_{23,1} * IKKIkBaNFkB\end{aligned} \tag{39}$$

$$\begin{aligned}\frac{\mathrm{d}}{\mathrm{d}t}IkBaNFkBn = & -\psi_{14,1} * IkBaNFkBn - \psi_{20,1} * IkBaNFkBn \\ & + (\psi_{13,1} * IkBaNFkB * \psi_{13,2}) + \psi_{18,1} * IkBan * NFkBn\end{aligned} \tag{40}$$

$$\frac{\mathrm{d}}{\mathrm{d}t}IkBat = \psi_5 + \frac{\psi_{6,1} * (NFkBn_{cas2}^{\psi_{6,2}})}{((NFkBn_{cas2}^{\psi_{6,2}}) + (\psi_{6,3}^{\psi_{6,2}}))} - \psi_{96,1} * IkBat \tag{41}$$

$$\begin{aligned}\frac{\mathrm{d}}{\mathrm{d}t}IKKIkBaNFkB = & -\psi_{23,1} * IKKIkBaNFkB - \psi_{25,1} * IKKIkBaNFkB \\ & + \psi_{21,1} * IKK * IkBaNFkB\end{aligned} \tag{42}$$

$$\frac{\mathrm{d}}{\mathrm{d}t}IKKIkBa = -\psi_{24,1} * IKKIkBa - \psi_{26,1} * IKKIkBa + \psi_{22,1} * IKK * IkBa \tag{43}$$

$$\begin{aligned}\frac{\mathrm{d}}{\mathrm{d}t}NFkB = & -\psi_{10,1} * NFkB - \psi_{17,1} * IkBa * NFkB - \psi_{95,1} * NFkB \\ & + (\psi_{12,1} * NFkBn * \psi_{12,2}) + \psi_{19,1} * IkBaNFkB + \psi_{25,1} * IKKIkBaNFkB\end{aligned} \tag{44}$$

$$\frac{\mathrm{d}}{\mathrm{d}t}NFkBn = -\psi_{12,1} * NFkBn - \psi_{18,1} * IkBan * NFkBn + (\psi_{10,1} * NFkB * \psi_{10,2}) + \psi_{20,1} * IkBaNFkBn \tag{45}$$

$$\frac{\mathrm{d}}{\mathrm{d}t}IKK_{off} = -\psi_{1,1} * stim * IKK_{off} - \psi_{2,1} * IKK_{off} - \psi_{67,1} * (TAK1^{\psi_{67,2}})/((TAK1^{\psi_{67,2}}) + (\psi_{67,3}^{\psi_{67,2}})) * IKK_{off} + \psi_{4,1} * IKK_i \tag{46}$$

$$\frac{\mathrm{d}}{\mathrm{d}t}IKK = -\psi_{3,1} * IKK - \psi_{21,1} * IKK * IkBaNFkB - \psi_{22,1} * IKK * IkBa + \psi_{1,1} * stim * IKK_{off} + \psi_{2,1} * IKK_{off} + \psi_{23,1} * IKKIkBaNFkB + \psi_{24,1} * IKKIkBa + \psi_{25,1} * IKKIkBaNFkB + \psi_{26,1} * IKKIkBa + \psi_{67,1} * (TAK1^{\psi_{67,2}})/((TAK1^{\psi_{67,2}}) + (\psi_{67,3}^{\psi_{67,2}})) * IKK_{off} \tag{47}$$

$$\frac{\mathrm{d}}{\mathrm{d}t}IKK_i = -\psi_{4,1} * IKK_i + \psi_{3,1} * IKK \tag{48}$$

2.8 Time delay (Core module)

$$\frac{\mathrm{d}}{\mathrm{d}t}IkBat_{cas1} = \psi_{96,1} * IkBat - \psi_{97,1} * IkBat_{cas1} \tag{49}$$

$$\frac{\mathrm{d}}{\mathrm{d}t}IkBat_{cas2} = \psi_{97,1} * IkBat_{cas1} - \psi_{98,1} * IkBat_{cas2} \tag{50}$$

$$\frac{\mathrm{d}}{\mathrm{d}t}NFkBn_{cas1} = \psi_{99,1} * NFkBn - \psi_{100,1} * NFkBn_{cas1} \tag{51}$$

$$\frac{\mathrm{d}}{\mathrm{d}t}NFkBn_{cas2} = \psi_{100,1} * NFkBn_{cas1} - \psi_{101,1} * NFkBn_{cas2} \tag{52}$$

2.9 Competition between CpG and polyIC

We use the hill equation formula to model the competition between CpG and polyIC. specifically, the CpG transported to endosome will reduce the cell capacity to transport polyIC, and vice versa. Thus, we multiply the endosomal transportation rate with the inhibition hill formula and replace the equations (24), (20) with the following two, respectively.

$$\frac{\mathrm{d}}{\mathrm{d}t}CpG_{en} = -\psi_{89,1} * CpG_{en} - \psi_{90,1} * CpG_{en,1} * TLR9 + \frac{\psi_{88,1} * CpG^{\psi_{88,3}}}{CpG^{\psi_{88,3}} + \psi_{88,2}^{\psi_{88,3}}} * \frac{\psi_{88,5}}{\psi_{88,7}^{\psi_{88,6}} + polyIC_{en}^{\psi_{88,6}}} + \psi_{91,1} * TLR9_{CpG} \tag{53}$$

$$\frac{\mathrm{d}}{\mathrm{d}t}polyIC_{en} = -\psi_{80,1} * polyIC_{en} - \psi_{81,1} * polyIC_{en,1} * TLR3 + \frac{\psi_{79,1} * polyIC^{\psi_{79,3}}}{polyIC^{\psi_{79,3}} + \psi_{79,2}^{\psi_{79,3}}} * \frac{\psi_{79,5}}{\psi_{79,7}^{\psi_{79,6}} + CpG_{en}^{\psi_{79,6}}} + \psi_{82,1} * TLR3_{polyIC} \tag{54}$$

2.10 notations

For simplicity, we denote all the molecular species with

$$x(t) = [x_1(t), ..., x_S(t)]^T = [stim(t), IkBa(t), ..., NFkBn_{cas2}(t)]$$

where each $x_i(t)$ corresponds to one species, S is the number of molecular species, t is the time. we denote all the chemical reaction parameters with

$$\psi = [\psi_{1,1}, \psi_{1,2}, ..., \psi_{1,n_1}, \psi_{2,1}, \psi_{2,2}, ..., \psi_{2,n_2}, ..., \psi_{L,1}, ..., \psi_{L,n_L}]$$

where $\psi_{j,k}, j = 1, ..., 101, k = 1, 2, 3, 4$ are parameters used in the equations (36) to (52). We postulate that cells share the structural model of gene expression as described above, but with varying parameters. The velocity of the change in concentration for each species can be described as a differential equation:

$$\dot{x}(t) = v(x(t), u(t), \psi), \tag{55}$$

where $u(t)$ is the input signaling of the system, and ψ as cell-specific parameters.

$$u(t) = \begin{cases} \text{ligand conc. vec} & t = 0 \\ 0 & \text{otherwise} \end{cases}$$

Our simulations with NFκB signaling model (equations (1) to (52)) begin with an equilibration phase (phase 0) that determines the concentrations of all species before $t_0 = 0$. right before the stimulus is received (phase 1). Phase 1 starts at $t_0 = 0$ when the stimulus is received, as the ligand concentration is directly added to the corresponding species of the system.

3 Scaling factor for NFκB standard units to arbitrary units

To compare the experimental measurement with the model simulation, converting the experimental observation Arbitrary Units to standard units is required. In this section, the ScaleFactor is defined as the scaling factor converting the units and defined by equation (56). To this end, we first estimated the cell volume and NFκB abundance, thus derive the theoretical maximal concentration of NFκB, which is corresponding to the observed maximum fluorescence of nuclear NFκB activity. The ScaleFactor is the number that convert the theoretical maximal NFκB concentration in standard units to experimental measured fluorescence in arbitrary units.

We measured the volume of a single macrophage, and it is approximately 6 pL (Measure by Dr. Stefanie Leucke). General reminder: $10^{-12}\,\text{L} = 1\,\text{pL}$.

Also, the nuclear volume may vary between cells. Swanson et al 1991 estimated that the nucleus vs cytoplasmic ratio for macrophages is 0.2. Adelaja et al. [1] used a value of 1/3.5 for the ratio of cytoplasmic vs nuclear volumes. In this work, a range of 2 – 6 fold for cytoplasmic vs nuclear volumes is assumed for giving a reasonable bounds of NFκB.

NFκB has been estimated to be 50,000 - 150,000 molecules per cell. Reminder: $1\,\text{M} = 6.022 \times 10^{23}$ molecules per L $= 6 \times 10^{11}$ molecules per pL. If we consider 60,000 molecules in a volume of 1pL that would correspond to 0.1μM.

In summary, (1) The volume of each abstract cell is 6pL; (2) the ratio of cytoplasmic and nuclear volume is ranged [2,6]; (3) NFκB has been estimated to be 50,000 - 150,000 molecules per cell. The theoretical maximum nuclear NFκB concentration ranges from $\frac{5\times10^4}{\frac{1}{1+2}\times 6\text{pL}} = 2.5 \times 10^4 pL^{-1} \approx 0.04\mu M$

to max-nuclei-NFκB-conc. $= \frac{15\times10^4}{\frac{1}{1+6}\times 6\text{pL}} = 1.75 \times 10^5 pL^{-1} \approx 0.3\mu M$. The theoretical cytoplasm NFκB concentration ranges from $\frac{5\times10^4}{\frac{6}{1+6}\times 6\text{pL}} = 0.97\times10^4 pL^{-1} \approx 0.016\mu M$ to $\frac{15\times10^4}{\frac{2}{1+2}\times 6\text{pL}} = 3.75\times10^4 pL^{-1} \approx 0.06\mu M$.

We then calculate the scaling factor transferring experimental observations of arbitrary units to model simulation standard units. For simplification, we assume (1) the bounds of the number of NFκB can be reached in any cell volume, (2) cells can achieve the highest concentration in response to stimuli (i.e. all NFκB can enter the nucleus), and we observe that (3) LPS-stimulated cells exhibit the highest response. To remove the influence of the outliers, the scaling factor is calculated as:

$$\text{ScaleFactor} = \frac{P_{0.9}(\{\max_{NF\kappa BrespondingtoLPS,t}\{NF\kappa B(t)\}\})}{\text{max-nuclei-NF}\kappa\text{B-conc.}} \tag{56}$$

where the $P_{0.9}(A)$ represents the 90% percentile of the set A, $NF\kappa B(t)$ is the experimental data of $NF\kappa B$ in macrophages responding to LPS. Then for our experiment data, the value of the scaling factor transferring A.U. to S.I. is 0.0313.

4 Mathematical formulation of signaling codons

In this part, we give the mathematical definition of the signaling codons we used in this study, which are based on Adelaja et al. [1]. duration (defined by (57)) measures the total time of NFκB level over a low threshold; time2HalfMaxPosInte (defined by (64)) measures how much NFκB activity is "front-loaded"; oscpower (defined by (63)) distinguishes oscillation features of trajectory; max-value (defined by (59)) and pos-pk1-amp (defined by (60)) measures the peak amplitude; max-pos-pk1-speed (defined by (62)), pos-pk1-time (defined by (61)), derivatives (defined by (58)), measures the activation speed; max-pos-integral (defined by (64)) measures the accumulated activity. We removed the metric of NumofPeaks, as the peak number of noisy experimental data is not comparable with the smooth model fitted data. We also removed the metric of peak2peak, as its measuring the difference between the highest and lowest value of a trajectory is redundant with the peak amplitude metric.

To calculate these metrics for noisy experimental cell trajectories, smoothing the trajectory is required. For an observed data sequence,

$$X^{(m)} = (x_1^{(m)}, x_2^{(m)}, x_3^{(m)}, ..., x_T^{(m)})$$

m is the cell index, and T is total time points, $x_i^{(m)}$ is the observed data of cell m at time point $i (= 1, 2, 3, ..., T)$. There are multiple way of smoothing the trajectories. The trajectory smoothed by a third-order one-dimensional median filter is represented by:

$$Y^{(m)} = \text{smooth}_{\text{med}}(X^{(m)}) = (y_1^{(m)}, y_2^{(m)}, y_3^{(m)}, ..., y_T^{(m)})$$

where $y_i^{(m)} = med(x_{i-1}^{(m)}, x_i^{(m)}, x_{i+1}^{(m)},)$. or smoothed by the mean filter:

$$Z^{(m)} = \text{smooth}_{\text{mean}}(Z^{(m)}) = (z_1^{(m)}, z_2^{(m)}, z_3^{(m)}, ..., z_T^{(m)})$$

where $z_i^{(m)} = mean(x_{i-1}^{(m)}, x_i^{(m)}, x_{i+1}^{(m)})$ or smoothed by Savitzky-Golay filter with 5-point quadratic polynomial:

$$S^{(m)} = \text{smooth}_{\text{sg}}(S^{(m)}) = (s_1^{(m)}, s_2^{(m)}, s_3^{(m)}, ..., s_T^{(m)})$$

where $s_i^{(m)} = 1/35(-3s_{i-2}^{(m)} + 12s_{i-1}^{(m)} + 17s_i^{(m)} + 12s_{i+1}^{(m)} - 3s_{i+2}^{(m)})$.

Based on these, we can define metrics duration, derivative, max-value as following

$$\text{duration}(Y^{(m)}) = \left|\left\{i : y_i^{(m)} > \lambda_d\right\}\right| \cdot \Delta t \tag{57}$$

$$\text{derivative}(Y_i^{(m)}) = (Y_{i+1}^{(m)} - Y_{i-1}^{(m)})/2\Delta t$$

The derivative feature is the value of derivative($Y_i^{(m)}$) at $i = 2$:

$$\text{derivative}(Y_2^{(m)}) = (Y_3^{(m)} - Y_1^{(m)})/2\Delta t \tag{58}$$

$$\text{max-value}^{(m)} = \max_i x_i^{(m)} \tag{59}$$

To define the peak-related metrics, N_D dominant peaks are picked out as described following. For trajectory $Z^{(m)}$, calculate the local maxima peak sets: $\Lambda_{\text{peak}} = \{z_i^{(m)} : z_i^{(m)} > z_{i-1}^{(m)}, z_i^{(m)} \geq z_{i+1}^{(m)}, z_i^{(m)} > 0\}$ and for peak $z_i^{(m)}$ define the dominant measurement by:

$$d(z_i^{(m)}) = \min\{z_i^{(m)} - \min_{j<i} z_j^{(m)}, z_i^{(m)} - \min_{k>i} z_k^{(m)}\}$$

The dominant peak set Λ_{dom} are composed of at most N_D dominant elements picked out from the Λ_{peak} such that:
(1) if $N_D < |\Lambda_{\text{peak}}|$,then $|\Lambda_{\text{dom}}| = N_D$ and $\inf \Lambda_{\text{dom}} \geq \sup \frac{\Lambda_{\text{peak}}}{\Lambda_{\text{dom}}}$.
(2) if $N_D \geq |\Lambda_{\text{peak}}|$, then $\Lambda_{\text{dom}} = \Lambda_{\text{peak}}$.

For features pos-pk1-amp, pos-pk1-time, max-pos-pk1-speed, the $N_D = 5$ dominant peaks are picked out, and the peak set is denoted as $\Lambda_{\text{dom},5}$. $i_{\text{peak1}}^{(m)}$ is the index of the smoothed data $Z^{(m)}$ where the first peak is reached with reaching time larger than $\text{Pos}_{\text{delay}} \cdot \Delta t = 3 \cdot \Delta t$.

$$i_{\text{peak1}}^{(m)} = \min_{i \in \Lambda_{\text{dom},5}, i>3} i$$

$$\text{pos-pk1-amp} = z_{i_{peak1}^{(m)}}^{(m)} \tag{60}$$

$$\text{pos-pk1-time}^{(m)} = (i_{peak1}^{(m)} - 1) \cdot \Delta t \tag{61}$$

To calculate the numerical maximal speed of the first peak, we first smooth the derivatives

$$\left(\text{derivative}(Y_1^{(m)}), \text{derivative}(Y_2^{(m)}), \text{derivative}(Y_3^{(m)}), ..., \text{derivative}(Y_T^{(m)})\right)$$

by local weighted scatterplot smoothing method (local weighted linear regression fit):

$$\text{smooth derivative}(Y_i^{(m)}) = \hat{a}_i Y_i^{(m)} + \hat{b}_i$$

where $\hat{a}_i$ and $\hat{b}_i$ is obtained from the local weighted linear least squares regression with window size span=5, and the cubic weight function $\omega(y) = (1 - dist(y, y_{focal})^3)^3$
`http://home.eng.iastate.edu/~shermanp/STAT447/Lectures/Cleveland%20paper.pdf`

$$\text{max-pk1-speed}^{(m)} = max_{j \leq i_{peak1}^{(m)}} \text{smooth derivative}(Y_j^{(m)}) \tag{62}$$

We utilize the spectral power calculated from Fourier transform to quantify the oscillation feature. The spectral power is increasing with the signal amplitude, which can obscure the oscillation feature of the

dynamics. Thus, to focus on the oscillation feature, we first normalize all the trajectories to the same amplitude then calculate the spectral power.

$$\text{ospower}^{(m)} = \sum_{k:0.33<f_k<1} \widehat{s^{(m)}}(f_k)^2 \tag{63}$$

$\widehat{s^{(m)}}(f_k)$ is the discrete Fourier transform of the signal $s_i^{(m)}$; f_k is the frequency and ranges from 0.33 hour^{-1} to 1 hour^{-1}; $\widehat{s^{(m)}}(f_k)^2$ is the power spectral density calculated by Fast Fourier Transform. We should note that this oscpower can distinguish the oscillatory trajectories and non-oscillatory trajectories, but not able to distinguish high-amplitude oscillation and low-amplitude oscillation, which can be made up by the peak amplitude codon. Finally, we dive into the integral-related metrics. Numerical integrals of original sequence $X^{(m)} int_i(X^{(m)}) = \sum_{k=1}^{i-1}(x_k^{(m)} + x_{k+1}^{(m)})\Delta t/2$ And the positive integrals of the original sequence $X^{(m)}$:

$$\text{posint}_i(X^{(m)}) = \sum_{k=1}^{i-1}((x_k^{(m)} + x_{k+1}^{(m)})^+ \Delta t)/2$$

where $(x_k^{(m)} + x_{k+1}^{(m)})^+$ is the positive part of $(x_k^{(m)} + x_{k+1}^{(m)})$.

$$\text{max-posint}(X^{(m)}) = max_{i=1,2,\ldots,N} \sum_{k=1}^{i-1}((x_k^{(m)} + x_{k+1}^{(m)})^+ \Delta t)/2 \tag{64}$$

Note that the

$$\text{time2HalfMaxPosIntegral} = \arg\min_i |\text{posint}_i(X^{(m)}) - \frac{1}{2}\max \text{posint}(X^{(m)})| \cdot \Delta t \tag{65}$$

5 Statistical Model

The experimental measurement is the fluorescence NFκB within the nucleus. To fit the experimental data for individual cells, we defined the output of the model as all possible formats of NFκB within the nucleus:

$$f(t, \psi) = (IkBaNF\kappa Bn(t, \psi) + NF\kappa Bn(t, \psi)) - \text{shift} \tag{66}$$

where $IkBaNF\kappa Bn(t, \psi)$ and $NF\kappa Bn(t, \psi)$ are calculated from the dynamical system (equations (1) to (52)) with the parameter values ψ. shift is mimicking the baseline deduction in processing the fluorescence image to deduct the background noise for each single cell.

We aim to estimate the parameters for individual cells by minimizing the "difference" between model simulation and experimental measurement. The following paragraphs explain how to achieve this step by step: 1) select parameters to be estimated; 2) introduce the individual-level statistical model to minimize the difference between exp. and sim; 3) introduce the population-level statistical model to constrain the individual parameter distribution. The third step is actually based on the assumption that all the cells are drawn from the same distribution, as they are genetically identical and treated the same way experimentally.

5.1 Parameter selection

As shown in the NFκB signaling model (equations (1) to (52)), there are more than 100 parameters in the model. To ensure the identifiability of the problem, and also considering the computational time, we only estimate a subset of ψ, i.e. only a few parameters were selected for fitting single-cell data.

For the IKK-NFκB common core module, four parameters governing the speed, magnitude, and oscillation of NFκB dynamics – TAK1 activation, total abundance of NFκB, and time-delay parameters of NFκB-regulated IκBα mRNA synthesis (Figure S1) – are selected. This selection is underpinned by experimental measurements exhibiting variability correlated with these parameters [1, 2, 14]. For receptor proximal modules, the variations in two or three key parameters – receptor synthesis, endosomal transport, deactivation of the signaling complex – that govern the pathway activation and deactivation are estimated (Figure S1). Their heterogeneity is supported by differentially expressed TLR/TNFR [5, 8, 21], variability in endosomal trafficking [6], and the complex degradation machinery [18]. These parameters were previously identified as potential drivers of cell-to-cell heterogeneity [6, 12].

As for the Mathematical notations, the parameters to be estimated include receptor synthesis rate ($\psi_{54,1}$ for TNF module, $\psi_{35,1}$ for LPS module, $\psi_{85,1}$ for CpG module, $\psi_{77,1}$ for PolyIC module, $\psi_{68,1}$ for Pam3CSK module), endosomal rate ($\psi_{36,1}$ for LPS module, $\psi_{88,1}$ for CpG module, $\psi_{79,1}$ for Pam3CSK module), degradation for receptor-ligand complex ($\psi_{58,1}$ for TNF module, $\psi_{44,1}$ for LPS module, $\psi_{93,1}$ for CpG module, $\psi_{83,1}$ for PolyIC module, $\psi_{75,1}$ for Pam3CSK module), TAK1 activation ($\psi_{52,2}$), time delay for NFκB regulated IKBa transcription ($\psi_{99,1}$, $\psi_{101,1}$), and abundance within a cell ($NF\kappa B(0)$). For simplification, we still donate the subset parameters for estimation as ψ.

5.2 Staistical model

We first introduce the **individual level (for single cell) statistical model**:

$$y_m(t) = f(t, \psi^{(m)}) + (a + f(t, \psi^{(m)})b)\varepsilon_m(t), \tag{67}$$

where the vector $y_m(t) \in \mathbb{R}^m$ is the observation of cell m over time (e.g., total nuclear NFκB concentration), m, $m = 1, 2, ..., M$. The function $f(t, \psi^{(m)})$ represents the NFκB signaling system output of the cell m. $\varepsilon(t) \sim \mathcal{N}(0, 1)$ is the white Gaussian noise, and $(a + f(t, \psi^{(m)})b)\varepsilon(t)$ involving both additive and multiplicative noise defines the error term modeling the difference between the observation and the true value. Here, a^2 and b^2 are the intensities of additive and multiplicative noise components, respectively.

For an observation at time t_i, we have

$$y_{mi} = f(t_i, \psi^{(m)}) + (a + f(t_i, \psi^{(m)}))\varepsilon_{mi}, \tag{68}$$

for cell $m = 1, \ldots, M$ and time points $i = 1, \ldots, T$. Experimental noise is assumed to be independent and identically distributed with $\varepsilon_{mi} \overset{\text{i.i.d.}}{\sim} \mathcal{N}(0, 1)$.

As all the experimentally measured cells have the same genome and are treated the same way, all the heterogeneous single cells are actually sampled from the same distribution. Therefore, instead of fitting each individual cell separately and independently, the parameters of individual cells $\psi^{(m)}$ are following a common **population-level statistical model**:

$$\phi^{(m)} = \log \frac{\psi^{(m)} - \psi_{lower}}{\psi_{upper} - \psi^{(m)}} \overset{\text{i.i.d.}}{\sim} \mathcal{N}(\mu, \Omega) \tag{69}$$

where μ is the fixed effects vector, representing the mean value of $\phi^{(m)}$. The random effects are the vector $\phi^{(m)} - \mu$ which are obtained from a multivariate Gaussian distribution with a mean of 0 and a covariance matrix Ω, which describes the deviation of individual parameters from μ. The logit function

applies a monotonic transformation to the parameters, resulting in non-normal parameter distributions with boundary vectors ψ_{lower} and ψ_{upper}. The vectors ψ_{lower} and ψ_{upper} are defined as 10^{-1} and 10^{1} fold change of the representative cell parameter values, respectively. For simplicity, the division of two vectors is the vector whose elements are the division of the corresponding elements from the two vectors.

5.3 Algorithms for MLE and MAP

The above statistical models (68) (69) is the nonlinear mixed effects model, with the latent variable (unobservable) ψ. The parameters to be identified for population level are $\theta = \{\mu, \Omega, a, b\}$. This can be done by maximum likelihood estimation (MLE).

As ψ is i.i.d. for different cells, and ε is i.i.d. for different cells, we have the likelihood function:

$$\mathcal{L}(\theta|Y) = p(Y|\theta) = \prod_{m=1}^{M} p(Y_m|\theta) \tag{70}$$

For cell m, we write the probability in formula with latent variable ψ:

$$p(Y_m|\theta) = \int p(Y_m, \psi^{(m)}|\theta)d\psi \tag{71}$$

The expectation-maximization (EM) algorithm can be applied to solve MLE with latent variable. Recall the EM algorithm:

E-step: calculate $Q(\theta|\theta_t) = \int log\left(p(Y, \psi|\theta)\right) p(\psi|Y, \theta_t)d\psi$

M-step: maximize $\theta_{t+1} = \arg\max_{\theta} Q(\theta|\theta_t)$

The following paragraphs show how to derive the log-likelihood for (69) with latent variable ψ.

$$p(Y_m, \psi^{(m)}|\theta) = p(Y_{m1}, Y_{m2}, ..., Y_{mT}, \psi^{(m)}|\theta) \tag{72}$$

$$Y_{mi} = f(t_i, \psi^{(m)}) + (a + f(t_i, \psi^{(m)})b)\varepsilon_{mi} \tag{73}$$

$$\varepsilon_{mi} \overset{\text{i.i.d.}}{\sim} \mathcal{N}(0, 1)$$

Given $\psi^{(m)}$, Y_{mi} is independent of Y_{mj} for $\forall i, j$.

$$p(Y_m, \psi^{(m)}|\theta) = p(Y_m|\psi^{(m)}, \theta)p(\psi^{(m)}|\theta) = p(\psi^{(m)}|\theta)\prod_{i=1}^{T} p(Y_{mi}|\psi^{(m)}, \theta) \tag{74}$$

To first calculate the $p(\psi^{(m)}|\theta)$, we define $\phi^{(m)} = g(\psi^{(m)}) = \log \frac{\psi^{(m)} - \psi_{lower}}{\psi_{upper} - \psi^{(m)}}$. Using chain rule, we have

$$\begin{aligned} p(\psi^{(m)}|\theta) &= p_{\phi}(g^{-1}(\psi^{(m)})|\theta) \\ &= \frac{1}{\sqrt{(2\pi)^k|\Omega|}} \exp\left(-\frac{1}{2}(\phi^{(m)} - \mu)^T\Omega^{-1}(\phi^{(m)} - \mu)\right)(g^{-1})'(\psi^{(m)}) \\ &= \frac{1}{\sqrt{(2\pi)^k|\Omega|}} \exp\left(-\frac{1}{2}(\phi^{(m)} - \mu)^T\Omega^{-1}(\phi^{(m)} - \mu)\right)\prod_{l=1}^{k} \frac{\psi_{upper,l} - \psi_{lower,l}}{\left(\psi_{upper,l} - \psi_l^{(m)}\right)\left(\psi_l^{(m)} - \psi_{lower,l}\right)} \end{aligned} \tag{75}$$

here $\psi_l^{(m)}$ is the lth element of $\psi^{(m)}$ vector, and we have in total k parameters in the ODE to be estimated for cell m. Then we want to calculate $p(Y_{mi}|\psi^{(m)}, \theta)$:

$$p(Y_{mi}|\psi^{(m)},\theta) = \frac{1}{\sqrt{2\pi}(a+f(t_i,\psi^{(m)})b)} \exp\left(-\frac{1}{2}\left(\frac{y_{mi}-f(t_i,\psi^{(m)})}{(a+f(t_i,\psi^{(m)})b)}\right)^2\right) \tag{76}$$

Given $\psi^{(m)}$, Y_{mi} is independent of Y_{mj} for $\forall i, j$, so we have

$$p(Y_m|\psi^{(m)},\theta) = \prod_{i=1}^{T} \frac{1}{\sqrt{2\pi}(a+f(t_i,\psi^{(m)})b)} \exp\left(-\frac{1}{2}\left(\frac{y_{mi}-f(t_i,\psi^{(m)})}{(a+f(t_i,\psi^{(m)})b)}\right)^2\right) \tag{77}$$

For (74), We substitute $p(Y_m|\psi^{(m)},\theta)$ with (77), and substitute $p(\psi^{(m)}|\theta)$ with (75), then we have:

$$\begin{aligned} p(Y_m,\psi^{(m)}|\theta) =& \frac{1}{\sqrt{(2\pi)^k|\Omega|}} \exp\left(-\frac{1}{2}(\phi^{(m)}-\mu)^T\Omega^{-1}(\phi^{(m)}-\mu)\right) \\ & \prod_{l=1}^{k} \frac{\psi_{upper,l}-\psi_{lower,l}}{\left(\psi_{upper,l}-\psi_l^{(m)}\right)\left(\psi_l^{(m)}-\psi_{lower,l}\right)} \\ & \prod_{i=1}^{T} \frac{1}{\sqrt{2\pi}(a+f(t_i,\psi^{(m)})b)} \exp\left(-\frac{1}{2}\left(\frac{y_{mi}-f(t_i,\psi^{(m)})}{(a+f(t_i,\psi^{(m)})b)}\right)^2\right) \end{aligned} \tag{78}$$

For different cells $m1$ and $m2$, the observations are independently identically distributed. Thus,

$$\begin{aligned} p(Y,\psi|\theta) &= \prod_{m=1}^{M} p(Y_m,\psi^{(m)}|\theta) \\ &= \prod_{m=1}^{M}\left(\frac{1}{\sqrt{(2\pi)^k|\Omega|}} \exp\left(-\frac{1}{2}(\phi^{(m)}-\mu)^T\Omega^{-1}(\phi^{(m)}-\mu)\right)\right) \\ & \prod_{m=1}^{M}\left(\prod_{l=1}^{k} \frac{\psi_{upper,l}-\psi_{lower,l}}{\left(\psi_{upper,l}-\psi_l^{(m)}\right)\left(\psi_l^{(m)}-\psi_{lower,l}\right)}\right) \\ & \prod_{m=1}^{M}\left(\prod_{i=1}^{T} \frac{1}{\sqrt{2\pi}(a+f(t_i,\psi^{(m)})b)} \exp\left(-\frac{1}{2}\left(\frac{y_{mi}-f(t_i,\psi^{(m)})}{(a+f(t_i,\psi^{(m)})b)}\right)^2\right)\right) \end{aligned} \tag{79}$$

Take the log of the above equation:

$$
\begin{aligned}
\log(p(Y,\psi|\theta)) = &- \sum_{m=1}^{M}\sum_{i=1}^{T}\log(a+f(t_i,\psi^{(m)})b) - \frac{1}{2}\sum_{m=1}^{M}\sum_{i=1}^{T}\left(\frac{y_{mi}-f(t_i,\psi^{(m)})}{a+f(t_i,\psi^{(m)})b}\right)^2 \\
&- \frac{k+T}{2}M\log(2\pi) - \frac{M}{2}\log(|\Omega|) - \sum_{m=1}^{M}\left(\frac{1}{2}(\phi^{(m)}-\mu)^T\Omega^{-1}(\phi^{(m)}-\mu)\right) \\
&+ M\sum_{l=1}^{k}\log(\psi_{upper,l}-\psi_{lower,l}) \\
&- \sum_{m=1}^{M}\sum_{l=1}^{k}\log(\psi_{upper,l}-\psi_l^{(m)}) \\
&- \sum_{m=1}^{M}\sum_{l=1}^{k}\log(\psi_l^{(m)}-\psi_{lower,l})
\end{aligned} \tag{80}
$$

The Expectation—maximization (EM) is an iterative algorithm to estimate the (local) maximum likelihood or maximum a posteriori (MAP) of parameters in statistical models involving unobserved latent variables. The algorithm iteratively calculate the expectation of the log-likelihood using the current estimate for the parameters (E step), and then computes parameters maximizing the expected log-likelihood calculated from the E step (M step). Stochastic Approximation Expectation Maximization (SAEM) provides a more robust and computationally efficient approach for EM by simulating the latent variables using the current parameter estimates and the available data [7, 10].

The SAEM algorithm iteratively refines the parameter estimates by gradually reducing the influence of the stochastic component, which allows for improved convergence properties compared to the standard EM algorithm. The algorithm is particularly useful when dealing with complex models or when the maximum likelihood estimates are difficult to obtain due to latent variables or non-linearities in the model.

After we estimated population-model parameters $\hat{\theta} = \{\hat{\mu}, \hat{\Omega}, \hat{a}, \hat{b}\}$, we then estimate the individual parameters ψ_m. We employ the Maximum a posteriori estimation (MAP), which is equivalent to estimating the condition mode of ψ_m given the prior distribution (estimated at the population level) and the corresponding single-cell data.

$$
\hat{\psi}^{(m)} = \arg\max_{\psi^{(m)}} p(\psi^{(m)}|Y_m;\hat{\theta}) \tag{81}
$$

This conditional probability can be rewritten using Bayesian law:

$$
p(\psi^{(m)}|Y_m;\hat{\theta}) = \frac{p(Y_m|\psi^{(m)};\hat{\theta})p(\psi^{(m)}|\hat{\theta})}{p(Y_m|\hat{\theta})} \tag{82}
$$

Here, $p(Y_m|\psi^{(m)};\hat{\theta})$ and $p(\psi^{(m)}|\hat{\theta})$ have the close form, and $p(Y_m|\hat{\theta})$ is a constant while to optimize over the space of $\psi^{(m)}$. With these closed forms, the objective function is a non-linear optimization problem, and the derivatives are unknown and difficult to compute due to the observation function being a function of the 52-dimensional-nonlinear ODE system solution. Thus, we applied the Nelder-Mead Simplex algorithm for this optimization problem in the multidimensional space without calculating the function derivatives formula. The initial value used for the Nelder-Mead simplex algorithm is the approximate conditional mean calculated at the end of the Stochastic Approximation Expectation-Maximization (SAEM) process, depending on availability. We employed the software Monolix for implementing these algorithms. see: `https://monolix.lixoft.com/` and `https://monolix.lixoft.com/tasks/ebes/`

To obtain the parameterization for each single cell, we first fit the core module parameter distribution to the TNF (representing oscillatory trajectories) and Pam (representing non-oscillatory trajectories) stimulated datasets. With the core module parameter distribution fixed to the estimated distribution from TNF and Pam stimulation, we then parameterize each receptor module using each ligand stimulation dataset. The parameters for each single cell are obtained from the MAP estimation. These single-cell parameters are then applied to simulate the corresponding NFκB signaling activities using the 52-dimensional ODE model. For TNF conditions, due to the poor recovery of noisy sustained oscillations, on the cells with simulated trajectories in the top 33% of the coefficient of variation (CV) were selected for further analysis, including evaluation of the model simulation and its applications.

6 Generate simulation data

6.1 Single ligand stimulation data

The fitted data can well capture the experimental data, and we want to expand the model scope to generate a new dataset that gives the same characteristic codon distributions as experimental data. As the inferred parameter distribution is an approximation of the true parameter distribution under the assumption of parameterized distribution, we first attempt to sample parameters from the estimated population statistic model:

$$\mathbf{logit}(\psi) \overset{\text{i.i.d.}}{\sim} \mathcal{N}(\mu, \Omega) \tag{83}$$

Then each sampled parameter set is applied to simulate the single-cell NFκB signaling activities using the 52-dimensional ODE of NFκB signaling network. The simulation results sampled from the parameterized distribution show a difference with the NFκB dynamic features fitted to the experiments (Supplementary Notes Figure S8).

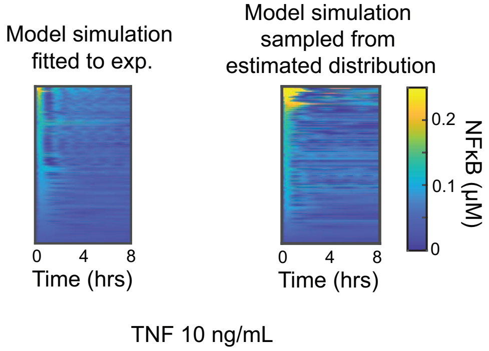

Figure S8. Model simulation fitted to experimental data vs model simulation run from sampled the parameter using the (83) sampling.

Thus, we take the bootstrapping approach, i.e. sampling from the non-parameterized discrete distribution:

$$f(\psi) = \sum_{m=1}^{M} \frac{1}{M} \delta(\psi - \hat{\psi}^{(m)}) \tag{84}$$

where, M is the total cell numbers for estimating individual parameters, and $\hat{\psi}^{(m)}$ from equation 81. The simulation results from this sampling approach are shown in Figure S3A in the manuscript, demonstrating consistency with experimental data.

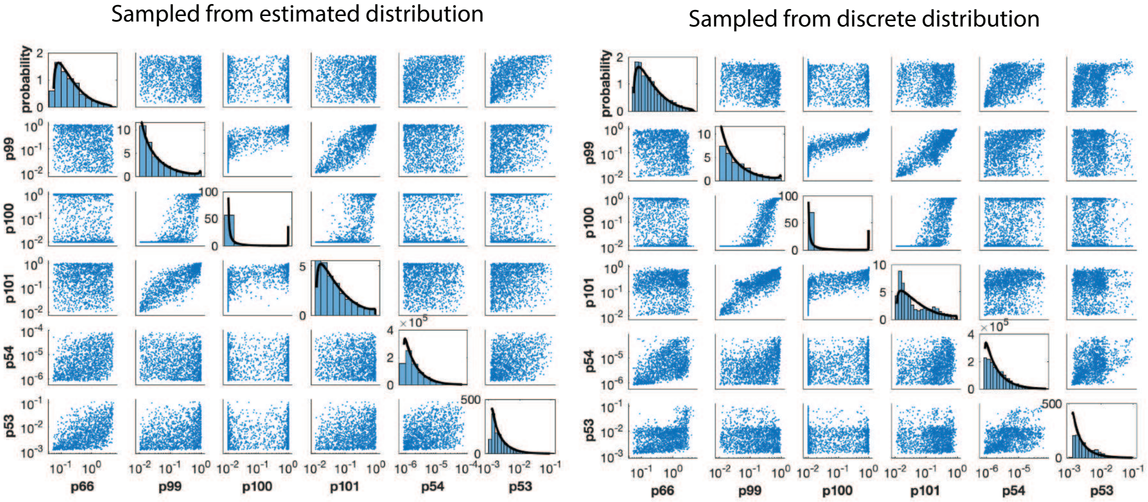

Figure S9. Parameter distribution of sampling from estimated distribution compared to sampling from the discrete distribution.

We then compared the generated distribution, in 1-D and 2-D projection space.

Bootstrapping Sampling approach (sampling from the discrete distribution composed of estimated parameter values) show a refinement distribution, as some parameter pairs with stronger correlation, and some parameter distributions with smaller parameter variance. Thus, the discrete parameter distribution is a refinement for the estimated population level logit normal distribution.

6.2 Dual-ligand stimulation data

For dual-ligand stimulation, we use specific rules to sample parameters, allowing us to simulate the NFκB trajectory and thus generate simulation data. Two rules guide the parameter sampling for dual-ligand stimulation, involving both the core module and two receptor modules:

1. For the first selected ligand-receptor module and core module, different parameter values are sampled from (84) using the bootstraping sampling apporach.

2. For the other ligand-receptor module parameters, they are sampled with a probability of one, having the minimal core module parameter distance from the first sampled core parameters.

Based on these two rules, we sample the parameters in two steps. The first step is to sample parameters for the core module and one of the two receptor modules. The second step is to match the other receptor module parameter based on the core module parameter similarity. These steps will be explained in the following paragraphs.

Step 1, sampling $[\psi_C, \psi_p]$ following the discrete distribution with probability mass function:

$$P([\psi_C, \psi_p] = [\psi_C^{(m)}, \psi_p^{(m)}]) = \frac{1}{M}$$

where $[\psi_C, \psi_p]$ is the vector R.V. in the parameter subspace corresponding to the core module (ψ_C) and the receptor module (ψ_p) responding to ligand p. m is the cell index, representing that parameter values are estimated from cell m. M is the total cell numbers for dataset in responding to ligand p. This step draws the sample uniformly from the parameter set estimated from ligand p stimulated dataset.

Step 2. is to match cells with similar core module parameters for datasets responding to ligand p and ligand q. Specifically, sampling $\psi_q^{(m)}$ with PMF:

$$P(\psi_q^{(m)} = \psi_q^{(n)}) = \begin{cases} 1 & \text{if } n = n_{(m,S_q)} \\ 0 & \text{otherwise.} \end{cases} \tag{85}$$

Here, parameter value $\psi_q^{(n)}$ are estimated from data of cell n responding to ligand q. $n_{(m,S_q)}$ is the cell whose core module parameters ψ_C are most similar with $\psi_C^{(m)}$ across all the cells responding to ligand q, i.e. for all $n \in S_q$. The detailed definition and calculation of $n_{(m,S_q)}$ will be explained in the following paragraph.

The IκB-NFκB core module is shared by all stimuli, so we will identify archetype cells in different stimulus conditions based on their core parameter values (Euclidean metric). More specifically, for sampled parameter $m \in S_p$ (estimated from data of cell m in response to stimulus p), we can find $n \in S_q$ (estimated from data of cell n in response to stimulus q) through the following optimization:

$$n_{(m,S_q)} = \underset{n \in S_q}{\operatorname{argmin}} \|\psi_C^{(n)} - \psi_C^{(m)}\|,$$

where $\psi_C^{(m)}$ represents the core parameter vector of cell m. Then we can extend the parameters of cell m for other receptor responding to stimulus q,

$$\tilde{\psi}_q^{(m)} = \psi_q^{(n_{(m,S_q)})},$$

where $\psi_q^{(n_{(m,S_q)})}$ represents the parameter of cell $n_{(m,S_q)}$ corresponding to receptor that responding to stimulus q; where $\tilde{\psi}_q^{(m)}$ represents the cell m's extended parameter vector with receptor q.

Given sample size N, we sample $\frac{N}{2}$ using both ligand p and q for first and second steps. After we got sampled parameters for core module and the two receptor modules, $[\psi_C^{(m)}, \psi_p^{(m)}, \psi_q^{(n_{(m,S_q)})}]$, we then simulated the corresponding ODE under the corresponding dual-ligand stimulation, p and q.

6.3 Multi-ligand stimulation data

For multi-ligand stimulation, we follow a workflow similar to that of dual-ligand stimulation. This involves the core module and receptor parameters $p_1, p_2, \ldots, p_z$.

The procedure is as follows:

1. Sample parameters for the core module and one of the receptors, just as in the dual-ligand stimulation workflow.

2. Sample parameters for the remaining receptors. This step is repeated until all parameters from the involved receptors are sampled.

Given a sample size N, we divide it equally among the ligands, resulting in $\frac{N}{z}$ samples for each ligand $p_1, p_2, \ldots, p_z$ in the first step. Step 2 is then repeated until all parameters for the remaining receptors are defined. Apply step 1 to a different receptor module and repeat until all modules are sampled. After completing the parameter sampling, we simulated NFκB signaling responses to the multi-ligand simulation using 52-dimensional ODE.

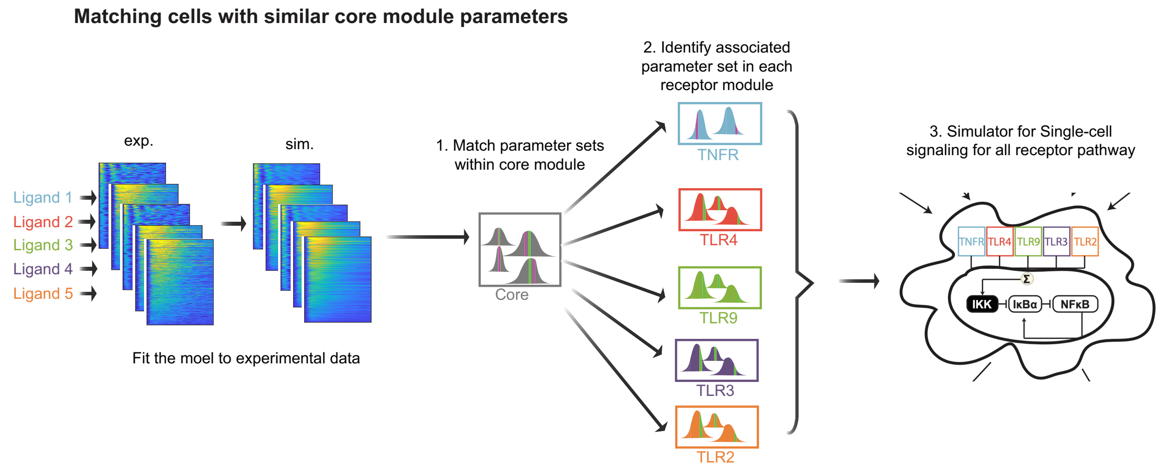

Figure S10. Workflow: Core module matching schematic presenting model's prediction procedure on 5 single ligand stimulation, achieved by matching sampled virtual cells with the most similar core module parameters.

6.4 Single-cell data responding to different ligands

The sampling approach in the last section "Multi-ligand stimulation data" is then leveraged for predicting single-cell NFκB activities to different stimulation. With the sampled parameters for all receptor and core module for each single cell, we simulate the 52-dimensional ODE system for different ligand stimulations (different initial values of Phase two). This way, we obtain z trajectories for each single cell, corresponding to the different ligand stimulations. This comprehensive approach allows us to capture the dynamic response of each cell to multiple ligands.

6.5 Test different matching methods

To simulate single-cell NFκB signaling responses to various stimuli, we considered the heterogeneity within the cell population, which stems from variations in parameter values both within the receptor modules and the common core module. Note that we have a collection of virtual single-cell NFκB signaling networks, each characterized by a set of core-module parameters and a set of parameters within one receptor module. The parameters were inferred from their corresponding experimentally stimulated single cells.

To generate single-cell responses to various stimuli, it is necessary to construct a comprehensive model of the virtual single cells, by incorporating the parameters of the core module and all five receptor modules. The association of specific receptor-associated parameter values with specific common core parameter values provides an opportunity to match the parameter values of two different receptor-associated modules based on the most similar core module parameter values (Supplementary Notes Figure S10). An essential criterion for an effective single-cell simulator capable of generating responses to various stimuli is accurately reproducing the population-level, stimulus-specific, and heterogeneous NFκB signaling responses observed in the single-ligand-stimulation experiments.

We compared the "Core Parameter Matching" (or "Similarity Matching") approach against "Random Matching", "Rescaled-Similarity Matching", and "Sampling Approximated Distribution". "Random Matching" of receptor module parameters using samples from inferred single-cell parameters; "Similarity Matching" based on the minimal Euclidean distance within the core module parameters (Supplementary Notes Figure S10); "Rescaled-Similarity Matching" using a rescaled (normalized to [0,1] for all parameters) Euclidean space for core module parameters; and "Sampling Approximated Distribution" by sampling

from an approximated logit-normal distribution of parameters, as opposed to using directly inferred parameters. Our analysis shows that the “Core Parameter Matching” most accurately captures the single-ligand-stimulated experimental signaling codon heterogeneous and stimulus-specific distributions.

7 Complex relationships between biochemical parameters and signaling codons

We first ordered the simulated NFκB trajectories by the rank of each of the six signaling codons, and depicted the corresponding parameter values for visual inspection (Figure not shown here due to size limit, available if requested). This revealed a complicated, uninterpretable pattern, prompting us to undertake a more in-depth correlations analysis.

We trained three regression models to understand the relationship between single-cell biochemical parameters (as inputs) and the signaling codons calculated from the corresponding simulated data (as outputs): (1) linear regression, (2) Random Forest regression, and (3) XGBoost (Extreme Gradient Boosting) regression. For each high-dose ligand stimulation, these regression models were trained using 67% of the data and tested using the remaining 33%. The accuracy scores of these models are evaluated via R-squared. The XGBoost regression, which showed the highest performance, was then used to calculate the feature importance of each biochemical parameter to quantify their contribution to the signaling codons. As negative controls, 10 random variables were generated for each single cell and included as additional inputs to the XGBoost regression to mimic non-correlated parameters or parameters that do not contribute to outputs. The regression models were implemented in Python, using the class ‘LinearRegression’ within the ‘sklearn.linear_model’ module of the ‘scikit-learn’ package, the class ‘RandomForestRegressor’ within the ‘sklearn.ensemble’ module of the scikit-learn package, and the class ‘XGBRegressor’ from ‘xgboost’ package.

The linear regression model yielded only low prediction accuracy, averaging 29%, with only 2 out of 30 signaling codons under different conditions achieving an accuracy greater than 60% (Supplementary Notes Figure S12A). The Random Forest model provided moderate performance, averaging 53%, with 10 out of 30 signaling codons achieving an accuracy over 60%. XGBoost showed slightly higher performance, averaging 67% with 24 out of 30 signaling codons exceeding 60% accuracy. These results imply that relationships between biochemical parameters and signaling codons are not linear.

To assess whether these relationships are monotonic, we calculated the Spearman correlations between signaling codons and parameters, but found them to be less than absolute 0.5 (Supplementary Notes Figure S12B): 30 out of 33 coefficients for Speed, 31/33 for Peak, 35/35 for Duration and Total, 29/33 for EvL, and 26/33 for Osc. Non-monotonicity is especially prominent in the signaling codons ‘Duration’ and ‘Total’, with the corresponding spearman correlation coefficients mainly concentrated in the range of less than 0.3 (Supplementary Notes Figure S13A). With the best, albeit modestly performing XGBoost model we asked whether we can identify specific biochemical parameters as determinants of NFκB signaling codon deployment. We calculated the feature importance, and 1/3 of the parameters rose above the 0.1 threshold that was generally not attained by random parameters (Supplementary Notes Figure S12C). However, only two parameters scored above 0.5 (TAK activation for Speed in response to Pam; signaling complex degradation for EvL in response to pIC; Supplementary Notes Figure S13B) indicating that generally the relationships between biochemical parameters and signaling codons are not only non-linear, non-monotonic but also highly multivariate.

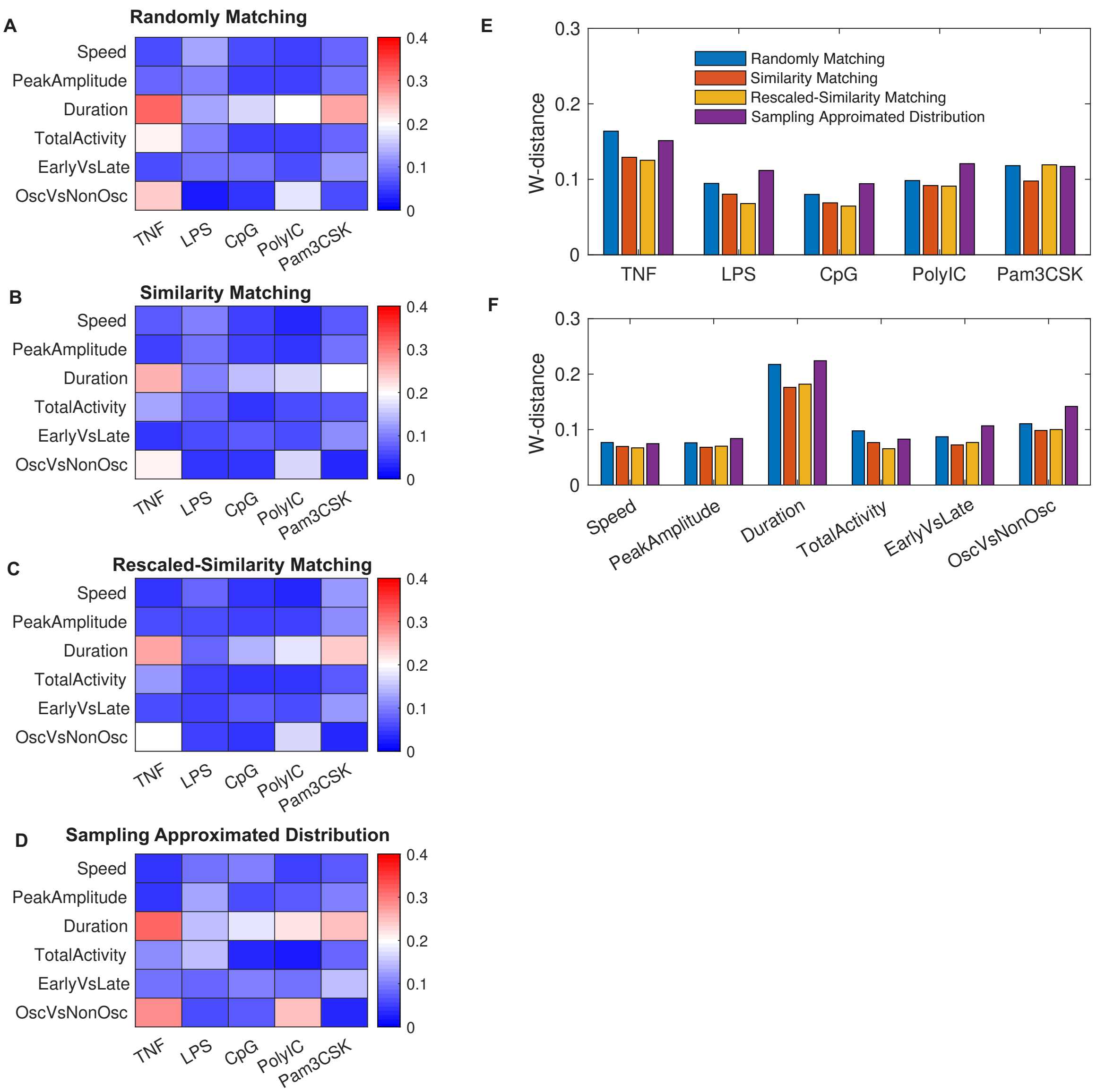

Figure S11. assessment of matching techniques for predicting single-cell responses to various ligand stimuli a-d. Heatmaps illustrating the Wasserstein distance between the signaling codon distributions predicted by the model and those observed in experiments. The analysis employs four distinct matching methods to align the five ligand-receptor module parameters: "Random Matching" (a), "Similarity Matching" (b), "Rescaled-Similarity Matching" (c), and "Sampling Approximated Distribution" (d). In the heatmaps, rows represent signaling codons, columns denote ligands, and the color intensity indicates the Wasserstein distance, providing a visual metric of similarity between model predictions and experimental data. e-f. Histogram of the average Wasserstein distance between the model-predicted and experimentally observed signaling codon distributions, summarized across signaling codons (e) and ligands (f).

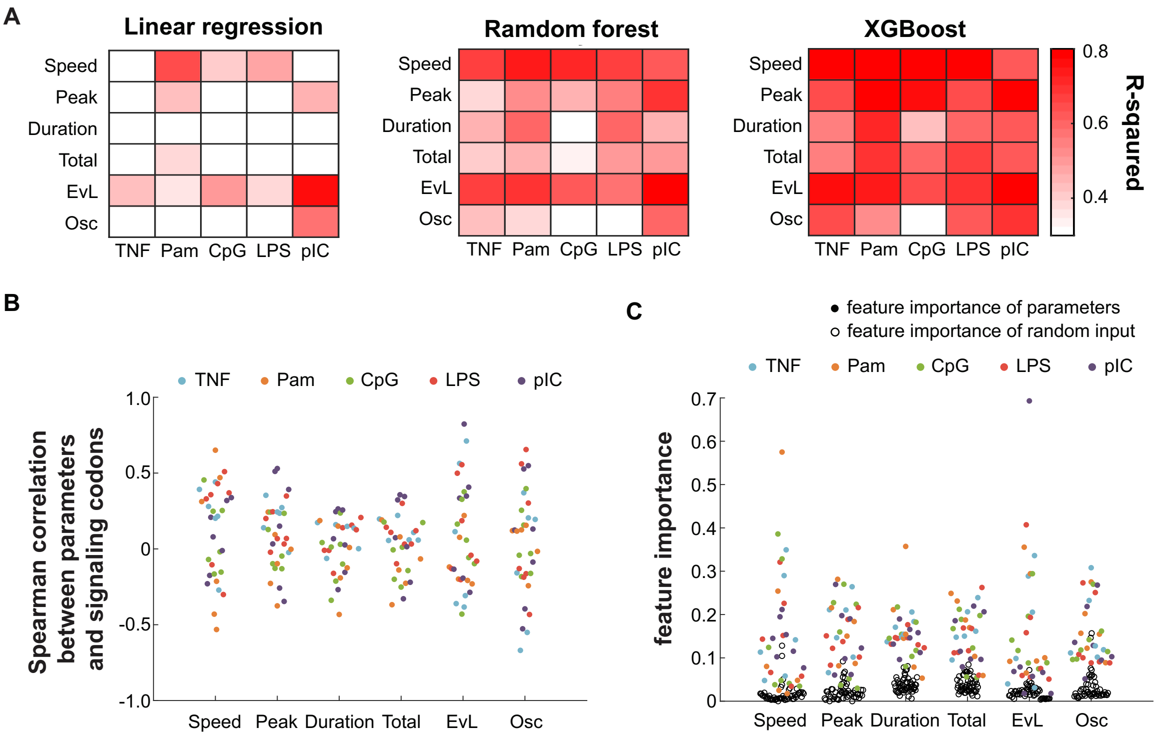

Figure S12. Examining the relationship between biochemical reaction rates and signaling responses (a) Heatmaps of the accuracy score (color bar on the right) for three different machine learning methods (specified in the title of each panel) that used the fitted parameter values as input and the corresponding calculated signaling codon as output (labeled on y-axis on the left) for different conditions (high dose of five ligands, labeled on the x-axis at the bottom). (b) Scatter plots illustrating the feature importance score of each parameter, as calculated via the XGBoost algorithm, using the fitted parameters (different filled dots) and 10 add-on parameters with randomly assigned values (hollow dots, as negative control) as input and the corresponding signaling codon (x-axis) as output for different conditions (high dose of five ligands, marked by different dot colors). (c) Scatter plots illustrating the Spearman correlation coefficients between fitted parameters (different dots) and model simulated signaling codons (x-axis) across five conditions (high dose of five ligands, marked by different dot colors).

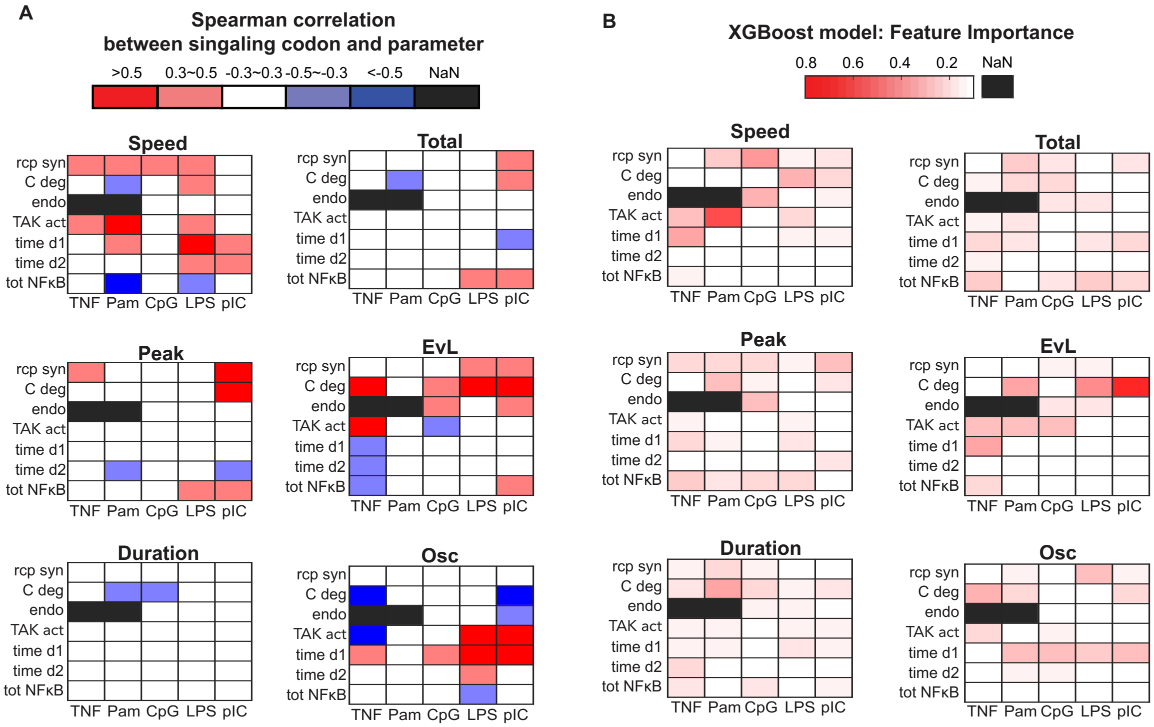

Figure S13. Relationships between biochemical reaction rates and NFκB signaling codons (a) Heatmaps of Spearman correlation coefficients between signaling codons and parameters for different stimulus conditions (columns). Each matrix focuses on the indicated Signaling Codon. Within each matrix, rows correspond to parameters, whose labeling are the same as in Figure S3A. (b) Heatmaps indicating the feature importance score of each parameter, as calculated via the XGBoost algorithm, using the fitted parameters as input and the indicated signaling codon as output for different conditions (high dose of five ligands, labeled on the x-axis at the bottom). The labeling are the same as in S12.

Supplementary References

1. Adewunmi Adelaja, Brooks Taylor, Katherine M Sheu, Yi Liu, Stefanie Luecke, and Alexander Hoffmann. Six distinct nfκb signaling codons convey discrete information to distinguish stimuli and enable appropriate macrophage responses. *Immunity*, 54(5):916–930, 2021.

2. James Bagnall, Christopher Boddington, Hazel England, Ruth Brignall, Polly Downton, Zainab Alsoufi, James Boyd, William Rowe, Alexander Bennett, Catherine Walker, et al. Quantitative analysis of competitive cytokine signaling predicts tissue thresholds for the propagation of macrophage activation. *Science signaling*, 11(540):eaaf3998, 2018.

3. Derren Barken, Chiaochun Joanne Wang, Jeff Kearns, Raymond Cheong, Alexander Hoffmann, and Andre Levchenko. Comment on "oscillations in nf-κb signaling control the dynamics of gene expression". *Science*, 2005.

4. Marcelo Behar and Alexander Hoffmann. Tunable signal processing through a kinase control cycle: the ikk signaling node. *Biophysical journal*, 105(1):231–241, 2013.

5. Chieh-Teng Cheng, Jye-Chian Hsiao, Alexander Hoffmann, and Hsiung-Lin Tu. Tnfr1 mediates heterogeneity in single-cell nf-κb activation. *Iscience*, 27(4), 2024.

6. Zhang Cheng, Brooks Taylor, Diana R Ourthiague, and Alexander Hoffmann. Distinct single-cell signaling characteristics are conferred by the myd88 and trif pathways during tlr4 activation. *Science signaling*, 8(385):ra69–ra69, 2015.

7. Bernard Delyon, Marc Lavielle, and Eric Moulines. Convergence of a stochastic approximation version of the em algorithm. *Annals of statistics*, pages 94–128, 1999.

8. Humberto M Garay-Malpartida, Roberta F Mourão, Marluce Mantovani, Icaro A Santos, Mari C Sogayar, and Anna C Goldberg. Toll-like receptor 4 (tlr4) expression in human and murine pancreatic beta-cells affects cell viability and insulin homeostasis. *BMC immunology*, 12:1–8, 2011.

9. Alexander Hoffmann, Andre Levchenko, Martin L Scott, and David Baltimore. The iκb-nf-κb signaling module: temporal control and selective gene activation. *science*, 298(5596):1241–1245, 2002.

10. Artémis Llamosi, Andres M Gonzalez-Vargas, Cristian Versari, Eugenio Cinquemani, Giancarlo Ferrari-Trecate, Pascal Hersen, and Gregory Batt. What population reveals about individual cell identity: single-cell parameter estimation of models of gene expression in yeast. *PLoS computational biology*, 12(2):e1004706, 2016.

11. Diane M Longo, Jangir Selimkhanov, Jeffrey D Kearns, Jeff Hasty, Alexander Hoffmann, and Lev S Tsimring. Dual delayed feedback provides sensitivity and robustness to the nf-κ b signaling module. *PLoS computational biology*, 9(6):e1003112, 2013.

12. Stefanie Luecke, Adewunmi Adelaja, Xiaolu Guo, Supriya Sen, Roberto Spreafico, Apeksha Singh, Yi Liu, Brooks Taylor, Jessica Diaz, Quen Cheng, et al. Tonic tnf conditioning of macrophages safeguards stimulus-specific inflammatory responses. *EMBO reports*, 24(7):e55986, 2023.

13. R Mosselmans, A Hepburn, Jacques Emile Dumont, Walter Fiers, and Paul Galand. Endocytic pathway of recombinant murine tumor necrosis factor in l-929 cells. *Journal of immunology (Baltimore, Md.: 1950)*, 141(9):3096–3100, 1988.

14. Yuji Ogura, Sajedah M Hindi, Shuichi Sato, Guangyan Xiong, Shizuo Akira, and Ashok Kumar. Tak1 modulates satellite stem cell homeostasis and skeletal muscle repair. *Nature Communications*, 6(1):10123, 2015.

15. D Shane O'Mahony, Uyenvy Pham, Ramesh Iyer, Thomas R Hawn, and W Conrad Liles. Differential constitutive and cytokine-modulated expression of human toll-like receptors in primary neutrophils, monocytes, and macrophages. *International journal of medical sciences*, 5(1):1, 2008.

16. Jangir Selimkhanov, Brooks Taylor, Jason Yao, Anna Pilko, John Albeck, Alexander Hoffmann, Lev Tsimring, and Roy Wollman. Accurate information transmission through dynamic biochemical signaling networks. *Science*, 346(6215):1370–1373, 2014.

17. Vincent F-S Shih, Jeffrey D Kearns, Soumen Basak, Olga V Savinova, Gourisankar Ghosh, and Alexander Hoffmann. Kinetic control of negative feedback regulators of nf-κb/rela determines their pathogen-and cytokine-receptor signaling specificity. *Proceedings of the National Academy of Sciences*, 106(24):9619–9624, 2009.

18. Kiyoshi Takeda, Tsuneyasu Kaisho, and Shizuo Akira. Toll-like receptors. *Annual review of immunology*, 21(1):335–376, 2003.

19. Sunao Takeshita, Keisuke Kaji, and Akira Kudo. Identification and characterization of the new osteoclast progenitor with macrophage phenotypes being able to differentiate into mature osteoclasts. *Journal of Bone and Mineral Research*, 15(8):1477–1488, 2000.

20. Naoki Watanabe, Hiroshi Kuriyama, Hisao Sone, Hiroshi Neda, Naofumi Yamauchi, Masahiro Maeda, and Yoshiro Niitsu. Continuous internalization of tumor necrosis factor receptors in a human myosarcoma cell line. *Journal of Biological Chemistry*, 263(21):10262–10266, 1988.

21. Jeff Werner, Correne A DeCarlo, Nicholas Escott, Ingeborg Zehbe, and Marina Ulanova. Expression of integrins and toll-like receptors in cervical cancer: effect of infectious agents. *Innate Immunity*, 18(1):55–69, 2012.

22. Shannon L Werner, Jeffrey D Kearns, Victoria Zadorozhnaya, Candace Lynch, Ellen O'Dea, Mark P Boldin, Averil Ma, David Baltimore, and Alexander Hoffmann. Encoding nf-κb temporal control in response to tnf: distinct roles for the negative regulators iκbα and a20. *Genes & development*, 22(15):2093–2101, 2008.

23. Ivan Zanoni, Renato Ostuni, Lorri R Marek, Simona Barresi, Roman Barbalat, Gregory M Barton, Francesca Granucci, and Jonathan C Kagan. Cd14 controls the lps-induced endocytosis of toll-like receptor 4. *Cell*, 147(4):868–880, 2011.
