# Supplementary material for: Modeling heterogeneous signaling dynamics of macrophages reveals principles of information transmission in stimulus responses
Source: Nat Commun. 2025 Jul 1;16:5986. doi: 10.1038/s41467-025-60901-3 (PMC12218168; doi:10.1038/s41467-025-60901-3)
Supplement: Supplementary file 2 — Description of Additional Supplementary Information [file 41467_2025_60901_MOESM2_ESM.pdf]

Description of Additional Supplementary Files:

Supplementary Data 1: the Species sheet lists each molecular species in the NFκB signaling network and its index used in the ODE simulations.

Supplementary Data 2: the Reactions sheet enumerates each biochemical reaction in the NFκB signaling network and its simulation index.
