# Supplementary material for: Modeling heterogeneous signaling dynamics of macrophages reveals principles of information transmission in stimulus responses
Source: Nat Commun. 2025 Jul 1;16:5986. doi: 10.1038/s41467-025-60901-3 (PMC12218168; doi:10.1038/s41467-025-60901-3)
Supplement: Supplementary file 4 — Reporting Summary [file 41467_2025_60901_MOESM4_ESM.pdf]

nature portfolio

Corresponding author(s): Alexander Hoffmann

Last updated by author(s): 03-24-2025

Reporting Summary

Nature Portfolio wishes to improve the reproducibility of the work that we publish. This form provides structure for consistency and transparency in reporting. For further information on Nature Portfolio policies, see our Editorial Policies and the Editorial Policy Checklist.

Statistics

For all statistical analyses, confirm that the following items are present in the figure legend, table legend, main text, or Methods section.

n/a	Confirmed	
☐	☒	The exact sample size (n) for each experimental group/condition, given as a discrete number and unit of measurement
☒	☐	A statement on whether measurements were taken from distinct samples or whether the same sample was measured repeatedly
☐	☒	The statistical test(s) used AND whether they are one- or two-sided<br>*Only common tests should be described solely by name; describe more complex techniques in the Methods section.*
☒	☐	A description of all covariates tested
☒	☐	A description of any assumptions or corrections, such as tests of normality and adjustment for multiple comparisons
☐	☒	A full description of the statistical parameters including central tendency (e.g. means) or other basic estimates (e.g. regression coefficient) AND variation (e.g. standard deviation) or associated estimates of uncertainty (e.g. confidence intervals)
☒	☐	For null hypothesis testing, the test statistic (e.g. F, t, r) with confidence intervals, effect sizes, degrees of freedom and P value noted<br>*Give P values as exact values whenever suitable.*
☒	☐	For Bayesian analysis, information on the choice of priors and Markov chain Monte Carlo settings
☒	☐	For hierarchical and complex designs, identification of the appropriate level for tests and full reporting of outcomes
☒	☐	Estimates of effect sizes (e.g. Cohen's d, Pearson's r), indicating how they were calculated

Our web collection on statistics for biologists contains articles on many of the points above.

Software and code

Policy information about availability of computer code

Data collection	The code used to tracking live-cell imaging and convert to time series data points is publicly available and has be deposited in GitHub at https://github.com/Adewunmi91/MACKtrack .
Data analysis	The code used to develop the model, perform the analyses and generate results in this study is publicly available and has been deposited in GitHub at https://github.com/Xiaolu-Guo/Virtual_single_cell_NFkB, under MIT license. The version v1.0.0 of the code associated with this publication is archived in Zenodo and is accessible via DOI: 10.5281/zenodo.15062580

For manuscripts utilizing custom algorithms or software that are central to the research but not yet described in published literature, software must be made available to editors and reviewers. We strongly encourage code deposition in a community repository (e.g. GitHub). See the Nature Portfolio guidelines for submitting code & software for further information.

Data

Policy information about availability of data

All manuscripts must include a data availability statement. This statement should provide the following information, where applicable:

- Accession codes, unique identifiers, or web links for publicly available datasets
- A description of any restrictions on data availability
- For clinical datasets or third party data, please ensure that the statement adheres to our policy

The experimental and simulation data generated in this study, and the processed data have been deposited in the Dryad database under DOI: 10.5061/dryad.8cz8w9h3d

Research involving human participants, their data, or biological material

Policy information about studies with human participants or human data. See also policy information about sex, gender (identity/presentation), and sexual orientation and race, ethnicity and racism.

Reporting on sex and gender	*Use the terms sex (biological attribute) and gender (shaped by social and cultural circumstances) carefully in order to avoid confusing both terms. Indicate if findings apply to only one sex or gender; describe whether sex and gender were considered in study design; whether sex and/or gender was determined based on self-reporting or assigned and methods used. Provide in the source data disaggregated sex and gender data, where this information has been collected, and if consent has been obtained for sharing of individual-level data; provide overall numbers in this Reporting Summary. Please state if this information has not been collected. Report sex- and gender-based analyses where performed, justify reasons for lack of sex- and gender-based analysis.*
Reporting on race, ethnicity, or other socially relevant groupings	*Please specify the socially constructed or socially relevant categorization variable(s) used in your manuscript and explain why they were used. Please note that such variables should not be used as proxies for other socially constructed/relevant variables (for example, race or ethnicity should not be used as a proxy for socioeconomic status). Provide clear definitions of the relevant terms used, how they were provided (by the participants/respondents, the researchers, or third parties), and the method(s) used to classify people into the different categories (e.g. self-report, census or administrative data, social media data, etc.) Please provide details about how you controlled for confounding variables in your analyses.*
Population characteristics	*Describe the covariate-relevant population characteristics of the human research participants (e.g. age, genotypic information, past and current diagnosis and treatment categories). If you filled out the behavioural & social sciences study design questions and have nothing to add here, write "See above."*
Recruitment	*Describe how participants were recruited. Outline any potential self-selection bias or other biases that may be present and how these are likely to impact results.*
Ethics oversight	*Identify the organization(s) that approved the study protocol.*

Note that full information on the approval of the study protocol must also be provided in the manuscript.

Field-specific reporting

Please select the one below that is the best fit for your research. If you are not sure, read the appropriate sections before making your selection.

[x] Life sciences   [ ] Behavioural & social sciences   [ ] Ecological, evolutionary & environmental sciences

For a reference copy of the document with all sections, see nature.com/documents/nr-reporting-summary-flat.pdf

Life sciences study design

All studies must disclose on these points even when the disclosure is negative.

Sample size	Each experimental condition yielded data on 176-744 cells as indicated in Table 1. The total number of cells (sample size) processed was constrained by the cell density in the culture and the number of regions that could be captured with a 5-minute imaging interval, then was determined by the algorithm MACKtrack (https://github.com/Adewunmi91/MACKtrack). No statistical method was used to predetermine the sample size. (See Statistics & reproducibility)
Data exclusions	Simulation dataset under TNF conditions, any model simulations exhibiting a low coefficient of variation (lowest 67% of CV) were excluded from further analysis, along with their corresponding experimental data, as they did not reliably capture the noisy sustained oscillations observed experimentally. (See Statistics & reproducibility)

Replication	Experimental data used for model fitting were representative of ten replicates, some of which were presented in previous publications 21,5,23,24. The TNF-stimulated samples in this study—without the soluble TNF receptor II—exhibited NFκB signaling dynamics consistent with previously published independent datasets, including Adelaja et al. 2021 (one biological replicate)21, Singh et al. 2024 (three biological replicates) 24, Rahman et al. 2024 (three biological replicates) 23, and Luecke et al. 2024 (two biological replicates) 5. This alignment with prior studies supports the reproducibility of the experimental work presented here.
Randomization	BMDMs were detached, re-plated, and randomly allocated to different wells (which will be applied to different stimulation). (See Experimental data generation)
Blinding	In our cell stimulation experiments, we assigned a coded numbering system to each sample tube to reduce awareness of sample identities during processing, thereby achieving a degree of blinding to group allocation. (See Experimental data generation)

Reporting for specific materials, systems and methods

We require information from authors about some types of materials, experimental systems and methods used in many studies. Here, indicate whether each material, system or method listed is relevant to your study. If you are not sure if a list item applies to your research, read the appropriate section before selecting a response.

Materials & experimental systems

n/a	Involved in the study
☒	☐ Antibodies
☒	☐ Eukaryotic cell lines
☒	☐ Palaeontology and archaeology
☐	☒ Animals and other organisms
☒	☐ Clinical data
☒	☐ Dual use research of concern
☒	☐ Plants

Methods

n/a	Involved in the study
☒	☐ ChIP-seq
☒	☐ Flow cytometry
☒	☐ MRI-based neuroimaging

Animals and other research organisms

Policy information about studies involving animals; ARRIVE guidelines recommended for reporting animal research, and Sex and Gender in Research

Laboratory animals	8-12 weeks old mVenus-RelA C57 Bl/6 endogenously-tagged mouse line (Adelaja et al., 2021)
Wild animals	N/A
Reporting on sex	Findings apply to both sexes. Experiments with either sex were undertaken and no differences were found.
Field-collected samples	N/A
Ethics oversight	The UCLA Institutional Animal Care and Use Committee approved the protocol for animal research per guidance from the American Veterinary Medical Association. All animal work complied with the IACUC policies and approved protocols.

Note that full information on the approval of the study protocol must also be provided in the manuscript.

Plants

Seed stocks	*Report on the source of all seed stocks or other plant material used. If applicable, state the seed stock centre and catalogue number. If plant specimens were collected from the field, describe the collection location, date and sampling procedures.*
Novel plant genotypes	*Describe the methods by which all novel plant genotypes were produced. This includes those generated by transgenic approaches, gene editing, chemical/radiation-based mutagenesis and hybridization. For transgenic lines, describe the transformation method, the number of independent lines analyzed and the generation upon which experiments were performed. For gene-edited lines, describe the editor used, the endogenous sequence targeted for editing, the targeting guide RNA sequence (if applicable) and how the editor was applied.*
Authentication	*Describe any authentication procedures for each seed stock used or novel genotype generated. Describe any experiments used to assess the effect of a mutation and, where applicable, how potential secondary effects (e.g. second site T-DNA insertions, mosiacism, off-target gene editing) were examined.*
